# Chiral-magic angle of nanoimprint meta-device

**DOI:** 10.1515/nanoph-2022-0733

**Published:** 2023-01-18

**Authors:** Mu Ku Chen, Jing Cheng Zhang, Cheuk Wai Leung, Linshan Sun, Yubin Fan, Yao Liang, Jin Yao, Xiaoyuan Liu, Jiaqi Yuan, Yuanhao Xu, Din Ping Tsai, Stella W. Pang

**Affiliations:** Department of Electrical Engineering, City University of Hong Kong, Kowloon, Hong Kong SAR, China; Centre for Biosystems, Neuroscience, and Nanotechnology, City University of Hong Kong, Kowloon, Hong Kong SAR, China; The State Key Laboratory of Terahertz and Millimeter Waves, City University of Hong Kong, Kowloon, Hong Kong SAR, China

**Keywords:** chirality, meta-device, metasurface, Moirépatterns, nanoimprint, twisted bilayer meta-devices

## Abstract

The magic angle of Twistronics has attracted a lot of attention because of its peculiar electrical characteristics. Moiré patterns formed by the superlattice of a twisted bilayer change overall physical properties. Circular dichroism can also be manipulated through the generated moiré pattern. Here, we report a polymer-based twisted bilayer meta-device fabricated by multilayer nanoimprint technology and study the magic angle of chirality. The superlattice of the bilayer meta-device creates moiré patterns and brings unique chiral optical responses. The bilayer nanoimprint technology is developed for metasurfaces with relative twist angles. Via the twist angle control, polymer materials with a low refractive index can manipulate the electric field of the light and reveal the chiral magic angle. Moreover, the shape of the meta-atoms plays a key role in chiral magic angle tuning. The chirality engineering by the reported nanoimprint technology and chiral meta-devices may contribute to applications in chiral imaging, biomedical sensing, lasing, and tunable optical devices.

## 1 Introduction

The magic angle of flat materials has attracted lots of attention because of the unconventional physical characteristics [1–3]. The superconductivity was created by stacking two graphene sheets with a small twist angle [4]. Photonic flat bands and tunable topological transitions were generated by the low-angle twisted bilayer photonic crystals or by mapping two layers into exactly one plane to gain strong coupling [5, 6]. In the latter case, the twist angle can be larger. Moiré patterns formed by the twisted double layers change overall physical properties [7]. In addition to the magic angles of superconductivity and photonic flat bands, the chiral magic angle is an essential phenomenon for physics as well. Circular dichroism can also be manipulated through the generated moiré pattern [8, 9]. Chiral materials are common in nature, such as various amino acids and proteins [10, 11]. They have a configuration that cannot overlap with their mirror symmetry structure [12]. However, the light–matter interaction resulting from chirality in natural materials is quite weak. The weak chiral signal limits the application of biomolecular detection and chiral light imaging [13, 14]. However, with artificial meta-atoms, chiral meta-devices have stronger chiral optical modulation efficiency than natural materials [15–17]. Different from the atomic arrangement of nature, meta-device can create extraordinary optical phenomena through artificial subwavelength structures with more degrees of freedom [18–21]. The geometry of meta-atoms design and their spatial arrangement can be manipulated to achieve significant light–matter interaction [22–26]. Therefore, meta-devices have been exploited for many applications, such as focusing [27–29], imaging [30–35], nonlinearity [36, 37], beam steering [38–40], computing [41], lasing [42], sensing [43, 44], polarization state generation and measurement [45, 46], vectorial optical fields generation [47], tunable devices [48–51], etc.

Most dielectric metasurfaces use high refractive index and low absorption loss materials for the light field localization and resonances [52–56]. It is a challenge to use common optical materials with low contrast of the refractive index, such as the widely used, highly transparent polymer material SU-8, a standard photoresist. As a soft material, SU-8 is an exemplary processing medium for nanoimprint technology [57–59]. Nanoimprint is a promising large-area fabrication technology for the industrial-scale mass production of meta-devices [60–62]. Through mechanical pressure and UV curing, nano/micro-structure patterns can be transferred from a mold to the target soft material using a stamping method [63–65]. With the technical advantages of ultra-high-resolution, easy mass production, low cost, and high reproducibility, nanoimprint technology makes it feasible to form large area of chiral optical modulation on low refractive contrast materials [66].

In this work, we report the circular dichroism and chiral magic-angle of the twisted bilayer meta-devices based on a low refractive index material (SU-8). The twisted bilayer meta-devices are fabricated by the advanced nanoimprint technology and precise stacking with rotation control. Applying SU-8 material and nanoimprint technology to the processing and research of chiral optics makes the realization of chiral meta-devices simpler, faster, and more precise. The bilayer meta-devices are stacked at certain twist angles and different moiré patterns are formed. This leads to different optical behaviors and chiral optical characteristics which can be observed theoretically and experimentally. The simulated electrical field of the twisted bilayer meta-devices has a good agreement with the signatures of the moiré pattern. The period of the moiré pattern matches the variation signature of the electric field with twisted angles. The optical responses of the twisted bilayer meta-devices are different under the left-hand circularly polarized (LCP) and right-hand circularly polarized (RCP) lights. The circular dichroism (CD) signal of the meta-device can be calculated using the experimental transmission spectra of the LCP and RCP light. The relative rotation angle can tune the chirality of the twisted bilayer meta-devices. The chiral magic angle is achieved at the twist angle where the CD signal is zero. With this chiral magic angle as the center, increasing or decreasing the twist angles by the same amount will have the opposite circular dichroism and anti-symmetry characteristics. This kind of physical critical point can provide unique optical chiral meta-devices for advanced meta-optics applications.

## 2 Results

**Figure 1:**
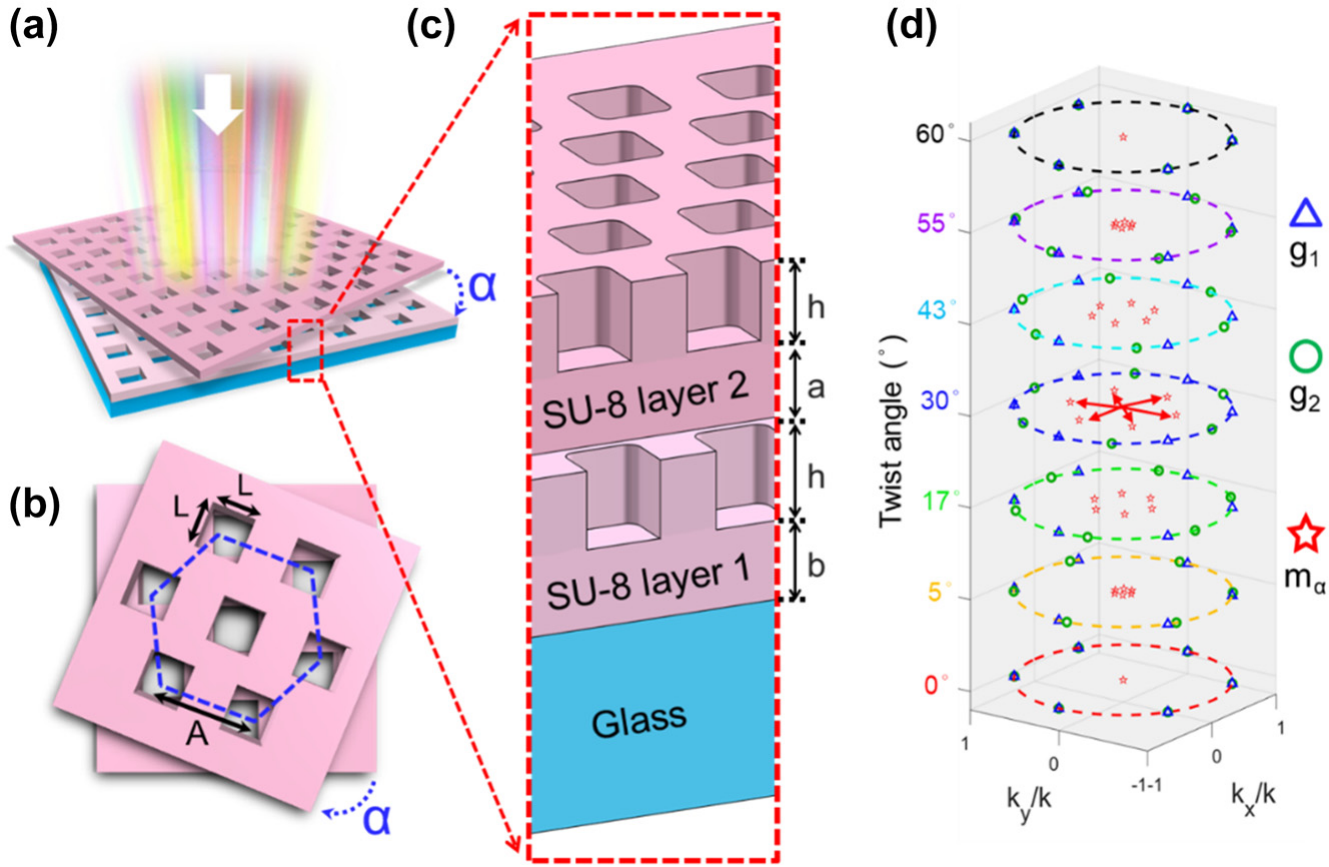
Schematic diagram of the twisted bilayer meta-devices. (a) Side view of the twisted bilayer meta-devices. “*α*” is the twist angle of the two layers of the meta-device. The illuminated light is a board band white light source. (b) Top view of the twisted bilayer meta-devices. “*L*” is 300 nm, which is the length of the square nano-hole of the meta-device. “*A*” is 535 nm, which is the period of the nano-holes, and the hexagonal blue dash line is the lattice arrangement. (c) The zoomed-in side view of the twisted bilayer meta-devices. “*h*” is 280 nm, which is the height of the nano-hole. “*a*” and “*b*” are 280 and 415 nm, which are the thicknesses of the top and bottom support layers, respectively. (d) Illustration of in-plane wave vectors of the twisted bilayer meta-devices with different twist angles. The blue triangles (*g*_1_) and green circles (*g*_2_) denote the reciprocal lattices of the two layers, respectively. The red arrows and red stars (*m*_*α*_) are the first-order moiré wave vectors, which are dependent on the twist angles shown on the *z*-axis. The dotted circles are the unit circle.

The twisted bilayer meta-device is composed of two layers of the SU-8 structured surfaces arranged in a hexagonal lattice with square holes, as shown in Figure 1(a). Figure 1(b) and (c) show the parameters setting of the twisted bilayer meta-device. The thickness of each layer of the twisted bilayer meta-device, h, is 280 nm. The side length (*L*) and period of the nano square hole (*A*) are 300 nm and 535 nm, respectively. The SU-8 support layer of the top layer metasurface, *a*, is set as 280 nm in thickness to support the top layer of nano-holes. The SU-8 support layer of the bottom metasurface, *b*, is set as 415 nm in thickness to strengthen adhesion to the underlying glass substrate (500 µm-thick). *α* is the twist angle of the twisted bilayer meta-device. The white arrow represents the incident light. Considering the scenario shown in Figure 1(a), an incident wave with an in-plane wave vector *k*_inc_ is scattered to a wave at *k*_inc_ + *g*_1_ upon transmission through the first layer. This transmitted wave should be scattered by the second layer into reflected and transmitted waves with in-plane wave vectors *k*_inc_ + *g*_1_ + *g*_2_. The reflected wave, in turn, should interact with the first layer, and the process would continuously iterate. At normal incidence, *k*_inc_ = 0. *g*_1_(*g*_2_) is the reciprocal lattice vectors of the first (second) layer in six different directions of this kind of hexagonal lattice, and their magnitude is |*g*_1_| = |*g*_2_| = $\frac{2\pi }{A}$ , where *A* is the period of the metasurface unit cell.

For incident light with an in-plane wave vector *k*_inc_, the modes in the twisted bilayer that are excited can be well described by the modes of a single photonic crystal slab coupled to a uniform dielectric slab with an incident in-plane wave vector at

(1) $$k_{\text{inc}}+m_{\alpha }=k_{\text{inc}}+g_{1}+g_{2}$$

where *m*_*α*_ = *g*_1_ + *g*_2_ is a moiré wave vector [67]. It should be noted that there should exist six different directions of *g*_1_ or *g*_2_ due to the hexagonal lattices. As a result, more than six *m*_*α*_ we should get. However, it is sufficient to consider only the lowest-order moiré wave vectors since we care more about the main resonance in our double-layer system. As shown in Figure 1(d), the red arrows and red stars are the first-order moiré wave vectors, which are sensitive to the twist angles *α*,

(2) $$\left|m_{\alpha }\right|=4\pi /A*\sin\left(\alpha /2\right)$$

This equation is consistent with the reciprocal lattice vectors of the moiré pattern observed in optical micrographs, which is *L* = *A*/(2*sin(*α*/2)). (The simulation details are shown in the simulation of method and Supporting Information.)

**Figure 2:**
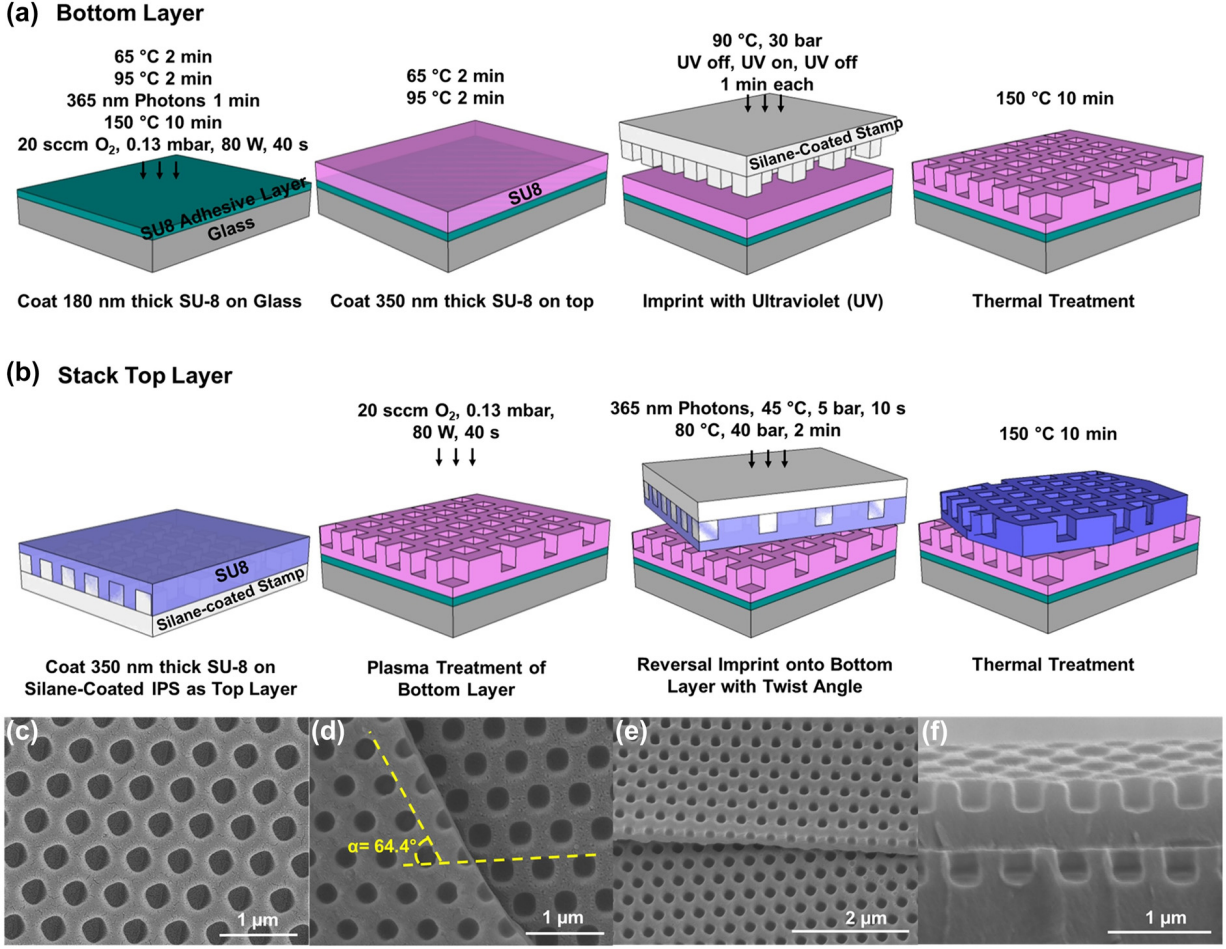
Fabrication technology and scanning electron micrographs for twisted bilayer meta-device. (a) Nano-holes in SU-8 polymer as the bottom layer were nanoimprinted with simultaneous UV exposure. (b) The top layer with nano-holes was reversal nanoimprinted onto the bottom layer with a twist angle. (c) Single polymer layer with nano-holes. (d) 64.4° twisted bilayer meta-device. (e) Tilted view of the bilayer meta-device in SU-8. (f) Cross-section of the bilayer meta-device with an intermediate layer.

The fabrication technology for the twisted bilayer meta-device is illustrated in Figure 2. A layer of SU-8 was deposited onto a cleaned glass substrate and was cured by ultraviolet (UV) light as an adhesive layer. After a subsequent layer of 350 nm thick SU-8 was deposited, a hexagonal array of square nano-holes with width, pitch, and depth of 300, 535, and 280 nm, respectively, was fabricated as the bottom layer of the bilayer photonic crystal by direct nanoimprint with a trichloro(1H,1H,2H,2H-perfluorooctyl)silane (FOTS)-coated intermediate polymer stamp (IPS), as shown in Figure 2(a). The fabrication of the top layer by reversal nanoimprint is shown in Figure 2(b). A 350 nm thick SU-8 layer was deposited onto an FOTS-coated IPS with pillars. This IPS with SU-8 was stacked upside down on top of the bottom layer with a twist angle. During reversal nanoimprint, UV was applied first to crosslink the top layer of SU-8 before the temperature and pressure were raised to 80 °C and 40 Bar to achieve good adhesion between the two layers. The IPS was demolded immediately after the reversal nanoimprint at 25 °C. The twisted bilayer meta-devices were completed by baking at 150 °C for 10 min. The structure of twisted bilayer meta-devices was examined by a scanning electron microscope. As shown in Figure 2(c), the photonic crystal slabs consisted of an array of uniform square nano-holes with a width of 300 nm and a pitch of 535 nm. Figure 2(d) shows the boundary between the top and bottom layers of a twisted bilayer meta-device with a twist angle of 64.4°. Twist angle was measured according to the orientations of the square nano-hole array with respect to each layer. The twist angle measurements from the scanning electron micrographs agreed with those conducted using the optical micrographs. In addition, Figure 2(e) shows a tilted view of the boundary for the twisted bilayer meta-device, showing a stack of two layers of nano-holes with an intermediate layer of SU-8 in between the nano-holes. The cross-section of the bilayer meta-device is revealed in Figure 2(f). The twisted bilayer meta-device consisted of 280 nm deep nano-holes on each layer, a 280 nm thick intermediate layer, and a 415 nm thick bottom layer.

**Figure 3:**
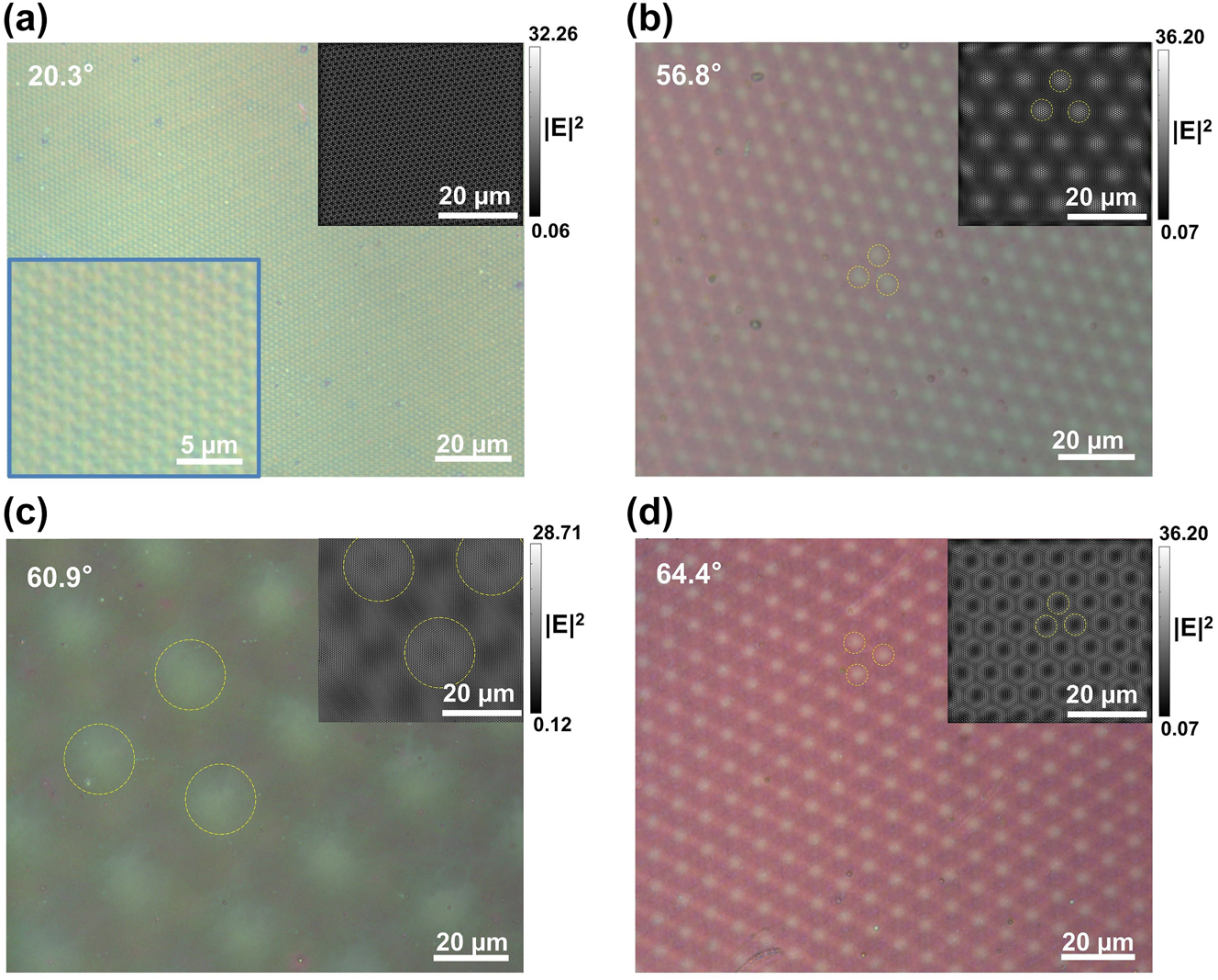
Optical micrographs and the simulated electric field distribution of moiré patterns. The twist angles are (a) 20.3°, (b) 56.8°, (c) 60.9°, and (d) 64.4°. The inserts show the simulated electric near-field distribution of the twisted bilayer meta-devices. The yellow dashed circles highlight the lattices of the measured moiré patterns and simulated electric near-field distribution.

Figure 3 shows the optical micrographs and the electric near-field simulation results of the twisted bilayer meta-devices with twist angles of 20.3°, 56.8°, 60.9°, and 64.4°, respectively. The moiré patterns generated by various twist angles of the twisted bilayer can be observed. The periods of the observed patterns are measured. The moiré pattern is expected to have a period provided by [68]:

(3) L=A/(2⁡sin(Δθ/2))

where *L* is the period of the moiré pattern, *A* is the period of the nano-holes, and Δ*θ* is the absolute degree difference between the twist angle and the closest axis of symmetry, namely, 0°, 60°, 120°, and so on. The calculated period of the moiré patterns with different twist angles is shown in Table S1. The measured moiré pattern period was found to be 1.4, 7.3, 35.1, and 8.0 µm for twist angles of 20.3°, 56.8°, 60.9°, and 64.4°, respectively. The inserts show the simulated electric near-field distribution of the twisted bilayer using Ansys Lumerical FDTD^®^, which looks very similar with the measured results. The yellow dashed circles highlight the lattices of the measured moiré patterns and simulated electric near-field distribution. The polarization state of the simulation model is LCP, and the analysis wavelength is 500, 535, 535, and 525 nm for the twist angle are 20.3°, 56.8°, 64.4°, and 60.3°, respectively. These wavelengths are used because they are one of the peaks of the respective CD signals.

**Figure 4:**
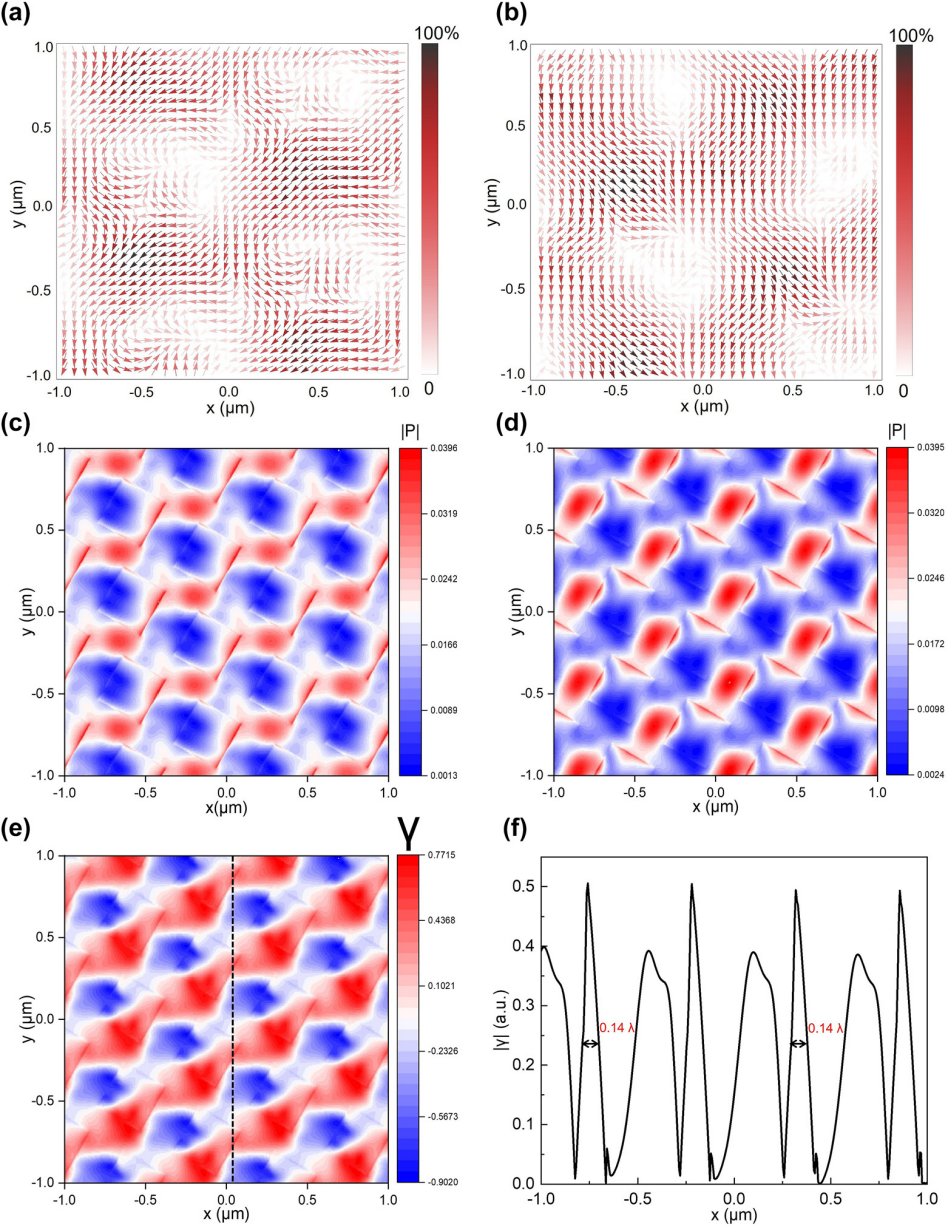
Light manipulation and subwavelength features by the twisted bilayer meta-devices. (a) and (b) The distribution of the Poynting vector at the surface of the twisted bilayer meta-device when the polarization states of the incident beam are LCP (a) and RCP (b), respectively. The wavelength of the normal incident light is 535 nm, and the twist angle of the meta-device is 65°. (c) and (d) The magnitude of the Poynting vector, *P*_LCP_, and *P*_RCP_, under the incidence of LCP beam (c) and RCP beam (d), when the twist angle is 60°. (e) The spatial variations of the magnitude of the Poynting vector versus the spin states are calculated as *γ* = (*P*_LCP_ − *P*_RCP_)/(*P*_LCP_ + *P*_RCP_). (f) A detailed view of the spatial variations across the black dashed line is shown in (e). The absolute value of the spatial variations |*γ*| is shown to facilitate the quantitative characterization of the feature size.

We found that even the twisted bilayer meta-devices made by the low refractive index contrast material, SU-8, can still effectively manipulate the incident light. For the incident wavelength of *λ* = 535 nm, Figure 4(a) and (b) show the distribution of Poynting vector at the surface of the twisted bilayer meta-device with a twist angle of 65° and the meta-device is illuminated by LCP and RCP beams, respectively. The direction of the arrow represents the direction of the Poynting vector, i.e., the power flow of the light field, and the color represents the magnitude. From the direction distributions and the color map of the Poynting vector, results showed the light field changed drastically when tuning the polarization state of the incident light. Figure 4(c) and (d) display the magnitude of the Poynting vector under two different circular polarizations, *P*_LCP_ and *P*_RCP_, when the twist angle is 60°. LCP and RCP illuminating results in different magnitudes of the Poynting vector distributions. The maximum value of energy flow is located at the right edge of the square hole under the LCP light illuminating. Under the illuminating RCP light, the maximum energy flow value is at the left/top edge of the square hole. This energy flow difference reveals links between far-field circular dichroism (spectral difference) and near-field modes’ perturbation. We calculated *γ* = (*P*_LCP_ − *P*_RCP_)/(*P*_LCP_ + *P*_RCP_) to find the spatial variations of the magnitude of the Poynting vector versus the spin states, as shown in Figure 4(e). Results display sub-wavelength features which are generated by the twisted bilayer meta-device. Figure 4(f) demonstrates that quantitative characterization of |*γ*| exhibits high spatial resolution features. The full width at half-maximum (FWHM) of the periodic features can be 0.14*λ*. When the twist angle deviates from this exotic angle (60°), the sub-wavelength features remain, but the periodicity will be changed. This subwavelength feature is determined by two parameters. The first parameter is the jump in the refractive index at the junction of the SU-8 and the air hole. This will lead to a hot spot at the junction [69]. The other parameter is the mode distribution related to the incident beam. Since the LCP and RCP beams will induce different mode distributions, the CD signal certainly has a close and complicated relationship with the subwavelength feature [70]. Results demonstrate that this twisted bilayer meta-device can effectively manipulate the incident light field and generate strong chirality with high spatial resolution. Figures S1 and S2 display similar results when the twist angles are 55° and 65°, respectively.

**Figure 5:**
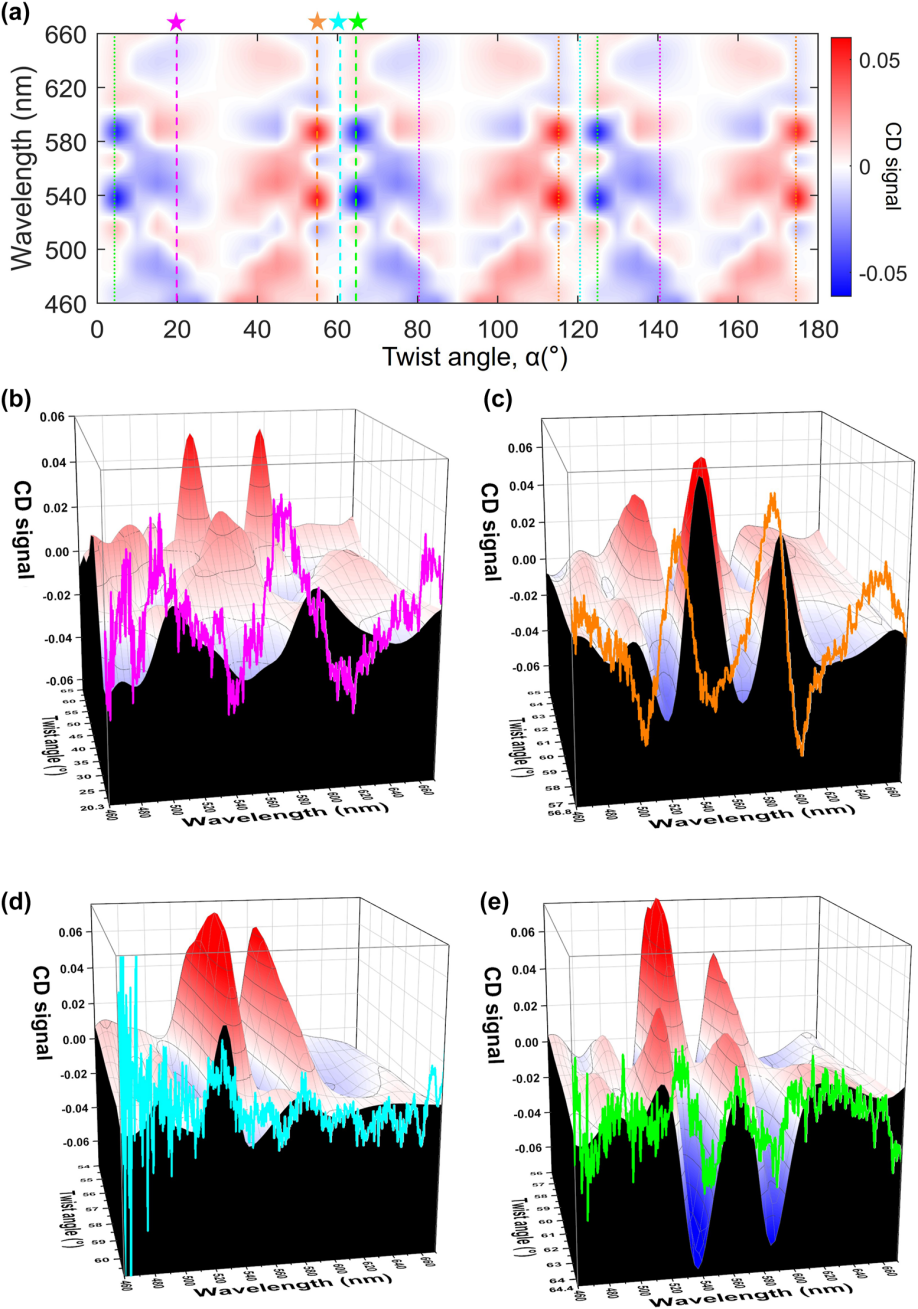
Circular dichroism (CD) of the twisted bilayer meta-devices. (a) Simulation of the CD signal map as a function of wavelength and twist angle. (b)–(e) The simulation and experimental CD signal for the twist angles of 20.3° (b), 56.8° (c), 60.9° (d), and 64.4° (e), respectively. The 3D distribution is the simulation results, and the black plan on edge is the simulation results of 20.3° (b), 56.8° (c), 60.9° (d), and 64.4° (e), respectively. The 2D colored curve is the experimental CD signal results of 20.3° (b), 56.8° (c), 60.9° (d), and 64.4° (e), respectively.

The simulated CD signal map of the twisted bilayer meta-devices is shown in Figure 5(a). The simulation model is verified, as shown in Figure S3. The transmission, *T*_LCP_ and *T*_RCP_ for the LCP and RCP incident beam are simulated and calculated. The wavelength range is from 460 nm to 670 nm, and the twist angles of the twisted bilayer meta-devices are set as 0°–180° with a 5° interval. We calculated the CD signal by Eq. (4).

(4) $$CD\;signal=\left(T_{\text{LCP}}-T_{\text{RCP}}\right)/\left(T_{\text{LCP}}+T_{\text{RCP}}\right)$$

Such normal transmission contrast of the incident circular polarization light in the far field by normal incidence shown in the map gives a measure of the strength of the CD signal. The CD signal repeats continuously and periodically with the twist angles, with a period of 60°. In every period, the CD signal is anti-symmetric. Among them, we selected four samples of different twist angles for verification of the CD map, i.e., 20.3°, 56.8°, 60.9°, and 64.4°, respectively. The theoretical values of the CD signal for these four samples are marked in this map with an asterisk. We also use dashed lines of the same color to identify the twist angles equivalent to them, i.e., the same CD signal due to the periodicity of the structure of the twisted bilayer meta-devices. Figure 5(b)–(e) show the experimental CD signal of the four samples. The optical measurement system is shown in Figure S4. The measured transmission was verified by the several samples and at various measured areas of the samples. Figures S5 and S6 show the simulated and experimental results of the single-layer meta-device and the twisted bilayer meta-device. In Figure 5(b)–(e), the curved part is the experimental result, and the 3D plot next to it is the theoretical value for a specific angular range near the experimental sample. These results demonstrate that the experimental signals and the simulation results match each other very well, and clearly show the trend of the CD signal. (Figure S7 shows the simulated and experimental CD signals in 2D colored curves) We also discussed the effect of the simulation area, the gap thickness, and the shape of the nanohole in Figure S8–S10, respectively.

**Figure 6:**
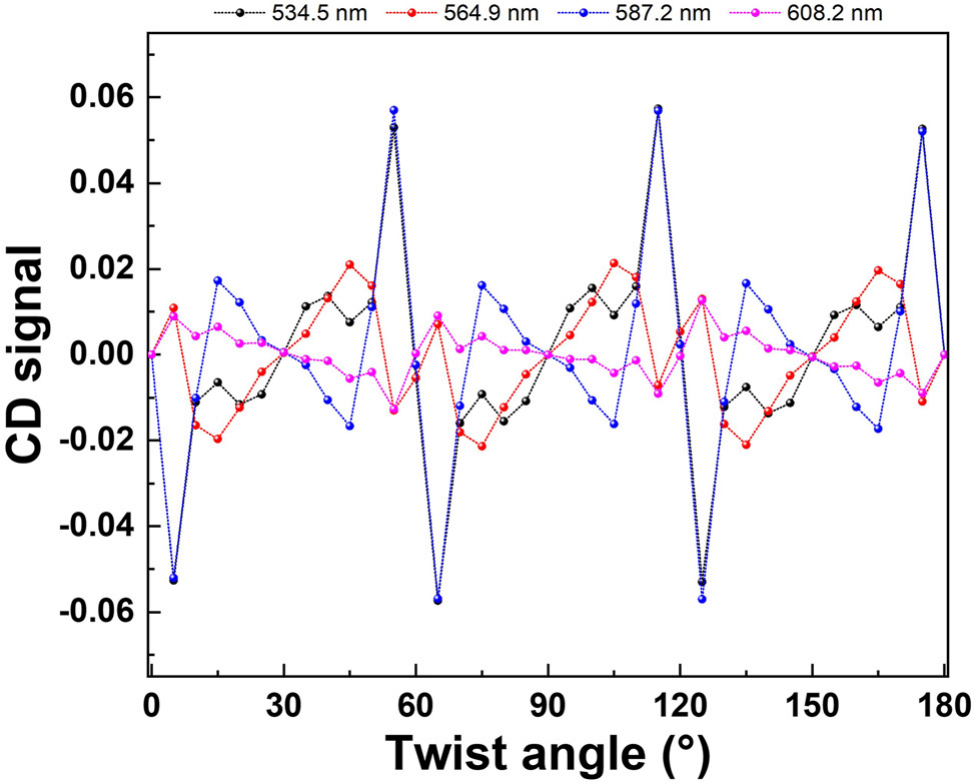
Chiral magic-angle of the twisted bilayer meta-devices. The orange lines are the merged points with zero CD signal, which is the chiral magic angles.

From the simulation results of the CD signal, we found that the CD signal is zero at 30°, 90°, and 150°, which means the *T*_LCP_ is equal to the *T*_RCP_. We determine these angles are the chiral magic angle. Figure 6 shows the CD signal values irradiated with four wavelengths of light at various twist angles. The four wavelengths are selected from the two peaks and two dips of the CD signal of the twisted bilayer meta-device with a 56.8° twist angle, as shown in Figure S11. The CD signals in all wavelengths at various twist angles are plotted in Figure S12. We can see that the plotted lines go through twist angles of 30°, 90°, and 150°. With this chiral magic angle as the center, increasing or decreasing the twist angles by the same amount will give the opposite chirality characteristics. At these chiral magic angles, the moiré patterns are already insensitive to the circular polarization of light. The moiré patterns are mirror-symmetrical when viewed from various axes of symmetry. The chiral optical response vanishes in the chiral magic angle. At 60° or 120° twist angles, the CD signals are slightly different. It is related to the shape of the meta-atom. The square-like nano-hole is anti-symmetric when the twisted bilayer meta-device has a 60° twist angle, which can be observed from the insert of Figure S12. When the meta-atom is a circular hole, the chiral magic angles appear at every 30° of twist angle, as shown in Figure S13. Compared with the square-like nano-hole of meta-atoms, we can see that the results of circular nano-hole show mirror symmetry when the twisted bilayer meta-device has a 60° twist angle. Therefore, the chiral magic angle can be tuned by the nano-hole shape of the bilayer meta-device.

**Figure 7:**
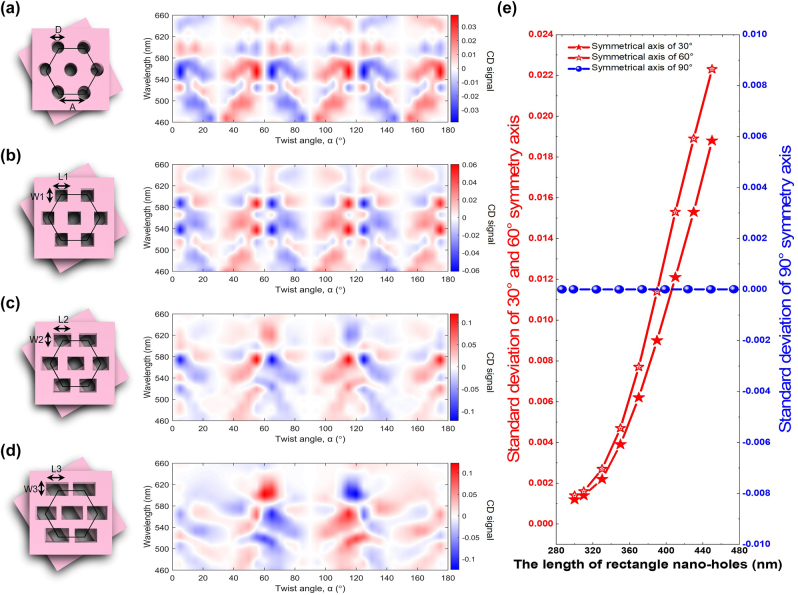
CD signal versus the shape of the unit cell. (a) Round holes with *D* = 300 nm. (b) Squared holes with *W*1 = *L*1 = 300 nm. (c) Rectangles with *W*1 = 300 nm and *L*2 = 370 nm. (d) Rectangles with one side length *W*3 = 300 nm and *L*3 = 430 nm. (e) The standard deviation of 30°, 60° and 90° symmetry axis versus the length of the rectangle nano-holes.

The effect of the shape and size of the meta-atoms on the CD signal can be found in Figure 7. Figure 7(a)–(d) show different shapes of meta-atoms and the corresponding CD signal maps. When the shape of the hole-like meta-atoms is changed from a circular hole to a rectangle hole with a relatively large aspect ratio, the symmetry of meta-atoms is gradually broken and hence the asymmetry of CD begins to increase. Figure 7(a) shows a circular hole with a diameter (*D*) of 300 nm as a meta-atom. Figure 7(b) shows a square hole with side length *L*1 = *W*1 = 300 nm. Figure 7(c) and (d) show meta-atoms of rectangular shapes, where the width, *W*2 and *W*3, both are 300 nm, and the lengths, *L*2 and *L*3, are 370 nm and 430 nm, respectively. The right column shows the corresponding CD signal maps. The horizontal axis is the twist angles, and the vertical axis is the wavelength range. From these four CD signal maps, the symmetry of the CD signal gradually evolves from every 30° to every 90° anti-symmetrically. The positions of chiral magic angle are changed due to the rotational asymmetry arising from the shape of the meta-atom changes from circular to rectangular. In addition to a visual representation of this trend in the CD maps, we also quantitatively describe this change. We calculate the standard deviation along with the mean values of 30°, 60°, and 90° twist angles and plot Figure 7(e) (see Figure S14-1 and S14-2). The horizontal axis is the length of the rectangle hole, and the vertical axis is the standard deviation of the symmetry axes. We found that the standard deviation of the 30° and 60° twist-angle symmetry axis becomes progressively larger, and the standard deviation of the 90° twist-angle symmetry axis stays around zero. When the meta-atom is a circular hole, magic angles emerge at 30°, 60°, and 90° twist-angle, similar to square-hole-like meta-atoms. However, when the symmetry of the meta-atom changes, the chiral magic angle of 30° and 60° disappears, and there is only a chiral magic angle of 90°.

## 3 Conclusions

We report the chiral optical response and chiral magic angle of the twisted bilayer meta-devices based on a low refractive index (*n* = 1.6) material (SU-8). We developed the advanced nanoimprint technology and precise stacking with rotation control for fabricating the twisted bilayer meta-devices. The twisted bilayer meta-device comprises two layers of square nano-hole meta-atoms arranged in a hexagonal lattice to form the moiré pattern. The chirality is measured and analyzed in good agreement with the numerical simulation. We found the twisted bilayer made by the low contrast index of refraction materials can effectively manipulate the power flow, and generate high spatial resolution (0.14 *λ*) features of the light field. The chirality of the twisted bilayer meta-devices can be tuned by the twist angle. Results showed an anti-symmetric chirality at the magic twist angle. With this chiral magic angle as the center, increasing or decreasing the twist angles by the same amount will have the opposite chirality characteristics. For three-fold symmetry meta-atoms, the magic angle can be found at 30°, 90°, 150°, 210°, 270°, and 330°, respectively. We found not only the shape but also the asymmetry of the meta-atoms introduces the change in the periodicity of the circular dichroism distribution and the chiral magic angle. We have successfully demonstrated advanced nanoimprint technology for the novel twisted bilayer meta-device with the technical advantages of easy mass production, high reproducibility, low cost, wide working bandwidth, visible light region, etc. This new chiral meta-device has great potential for the applications of biomedical molecule sensing, chiral imaging, quantum emitter, spin lasing, and tunable optical devices.

## 4 Methods

**Simulation** A three-dimensional finite-difference-time-domain method (Ansys Lumerical FDTD^®^) is used to simulate the near-field distribution and the transmission characteristics of the twisted bilayer meta-devices. We apply the perfect matching layer (PML) conditions in all directions to avoid boundary reflections. The simulation area is 20 µm * 20 µm. The conformal mesh with spatial resolution is less than 1/25 of the smallest feature size. The refractive index of the glass substrate is set as that in the Material Database, and the refractive index of SU-8 is set as 1.6 for the selected spectrum. For the model of twisted bilayer meta-devices, the twist angle is tuned by rotating the top layer and keeping the bottom layer the same.

**Fabrication** Figure 2(a) and (b) show the fabrication technology for the twisted bilayer meta-devices. The bottom layer of the bilayer photonic crystal was fabricated by direct nanoimprint with an IPS onto 350 nm thick SU-8 polymer according to method [58]. A 0.5 mm thick glass slide was first cleaned with acetone, isopropanol, and deionized water to remove the organic residue on the surface. Next, a plasma treatment was applied to make the surface hydrophilic and to clean the surface more thoroughly. A two-step plasma treatment was used, including 500 sccm N_2_ at 0.15 mbar and 300 W for 10 min and 200 sccm O_2_ at 0.15 mbar and 300 W for 20 min. To form the bottom layer as shown in Figure 2(a), a layer of 180 nm thick SU-8 was deposited onto the glass and baked at 65 °C and 95 °C for 2 min each. This SU-8 layer was crosslinked by 1 min exposure of 365 nm UV light as an adhesive layer, followed by hard baking at 150 °C for 10 min. On top of the adhesive layer, O_2_ plasma was applied with 20 sccm O_2_ and 80 W at 0.13 mbar for 40 s, and then a layer of 350 nm thick SU-8 polymer was coated. It was then baked at 65 °C and 95 °C for 2 min each. An IPS with nanopillars was coated with FOTS at 80 °C for 30 min so that the stamp could be separated from the SU-8 polymer after nanoimprint [60]. The 350 nm thick SU-8 layer was imprinted with the silane-coated IPS at 90 °C with 30 bar for 1 min, followed by UV exposure at 40 mW, 90 °C, and 30 bar for 1 min. The nanoimprint was completed with an additional 1 min of imprinting at 90 °C and 30 bar with UV off. The IPS was demolded and baked at 150 °C for 10 min, resulting in a hexagonal array of square nano-holes with width, pitch, and depth of 300 nm, 535 nm, and 280 nm, respectively.

Figure 2(b) shows a twisted bilayer meta-device fabricated by the reversal nanoimprint technique described in our previous work [59, 61, 62, 66]. A 350 nm thick SU-8 layer was deposited onto the FOTS-coated IPS with pillars. Meanwhile, an O_2_ plasma was applied onto the bottom layer with 20 sccm O_2_ and 80 W at 0.13 mbar for 40 s to increase the surface energy [63]. The IPS with the SU-8 coating as the top layer was turned upside down and stacked on top of the bottom layer with a twist angle [64]. Reversal nanoimprint was carried out with 365 nm photons at 45 °C and 5 Bar for 10 s to crosslink the top layer of SU-8. After the UV exposure, the temperature and pressure were raised to 80 °C and 40 Bar for 2 min to provide good adhesion of the top layer onto the bottom layer [65]. The IPS was demolded immediately after the reversal nanoimprint at 25 °C. The twisted bilayer meta-devices were completed by baking at 150 °C for 10 min.

## Supplementary Material

Supplementary Material Details
