# Supplementary material for: Chiral-magic angle of nanoimprint meta-device
Source: Nanophotonics. 2023 Jan 18;12(13):2479–90. doi: 10.1515/nanoph-2022-0733 (PMC11501801; doi:10.1515/nanoph-2022-0733)
Supplement: Supplementary file 1 — Supplementary Material Details [file j_nanoph-2022-0733_suppl.docx]

**Supporting Information of “Chiral-Magic Angle of Nanoimprint Meta-device”**

Mu Ku Chen^1,2,3,†^, Jing Cheng Zhang^1,3,†^, Cheuk Wai Leung^1,2,†^, Linshan Sun^1^, Yubin Fan^1^, Yao Liang^1^, Jin Yao^1^, Xiaoyuan Liu^1^, Jiaqi Yuan^1^, Yuanhao Xu^1,2^, Din Ping Tsai^1,2,3,*^, and Stella W. Pang^1,2,3,*^

^1^Department of Electrical Engineering, City University of Hong Kong, Kowloon, Hong Kong SAR, China

^2^Centre for Biosystems, Neuroscience, and Nanotechnology, City University of Hong Kong, Kowloon, Hong Kong SAR, China

^3^The State Key Laboratory of Terahertz and Millimeter Waves, City University of Hong Kong, Kowloon, Hong Kong SAR, China

†These authors contributed equally to this work.

*** Corresponding Authors: [dptsai@cityu.edu.hk](mailto:dptsai@cityu.edu.hk) (D.P.T.); [pang@cityu.edu.hk](mailto:pang@cityu.edu.hk) (S.W.P.)

**This word file includes:**

**Section 1: Effective manipulation of the light field**

Figure S1 Light manipulation and subwavelength features of the twisted bilayer meta-devices.

Figure S2 Light manipulation and subwavelength features of the twisted bilayer meta-devices.

**Section 2: Period of the moire pattern**

Table S1 Periods of the moiré patterns of the twisted bilayer meta-devices with different twist angles.

**Section 3: Simulation mode verification and experimental setup**

Figure S3 Simulation of the single-layer sample.

Figure S4 Experimental setup of the spectrum measurement.

Figure S5 Experimental results of the single-layer sample.

Figure S6 Experimental results of twisted bilayer meta-device.

Figure S7 Simulation and experimental results of CD signals.

Figure S8 The experimental and simulated CD signals of the meta-device with the twist angle of 60.9°.

Figure S9 Simulated CD signals versus different distances between two slabs when the twist angle is 56.8°.

Figure S10 CD signals when the twist angle of the meta-device is 20.3°.

**Section 4 : Chiral magic angle**

Figure S11 Selected wavelength for the figure plotting of the chiral magic angle.

Figure S12 Chiral magic angle of the square-like nano-hole of the twisted bilayer meta-device.

Figure S13 Chiral magic angle of the circular-like nano-hole of the twisted bilayer meta-device.

Figure S14 Chiral magic angle of the twisted bilayer meta-device with various shapes of meta-atoms.

**Section 1: Effective manipulation of the light field.**


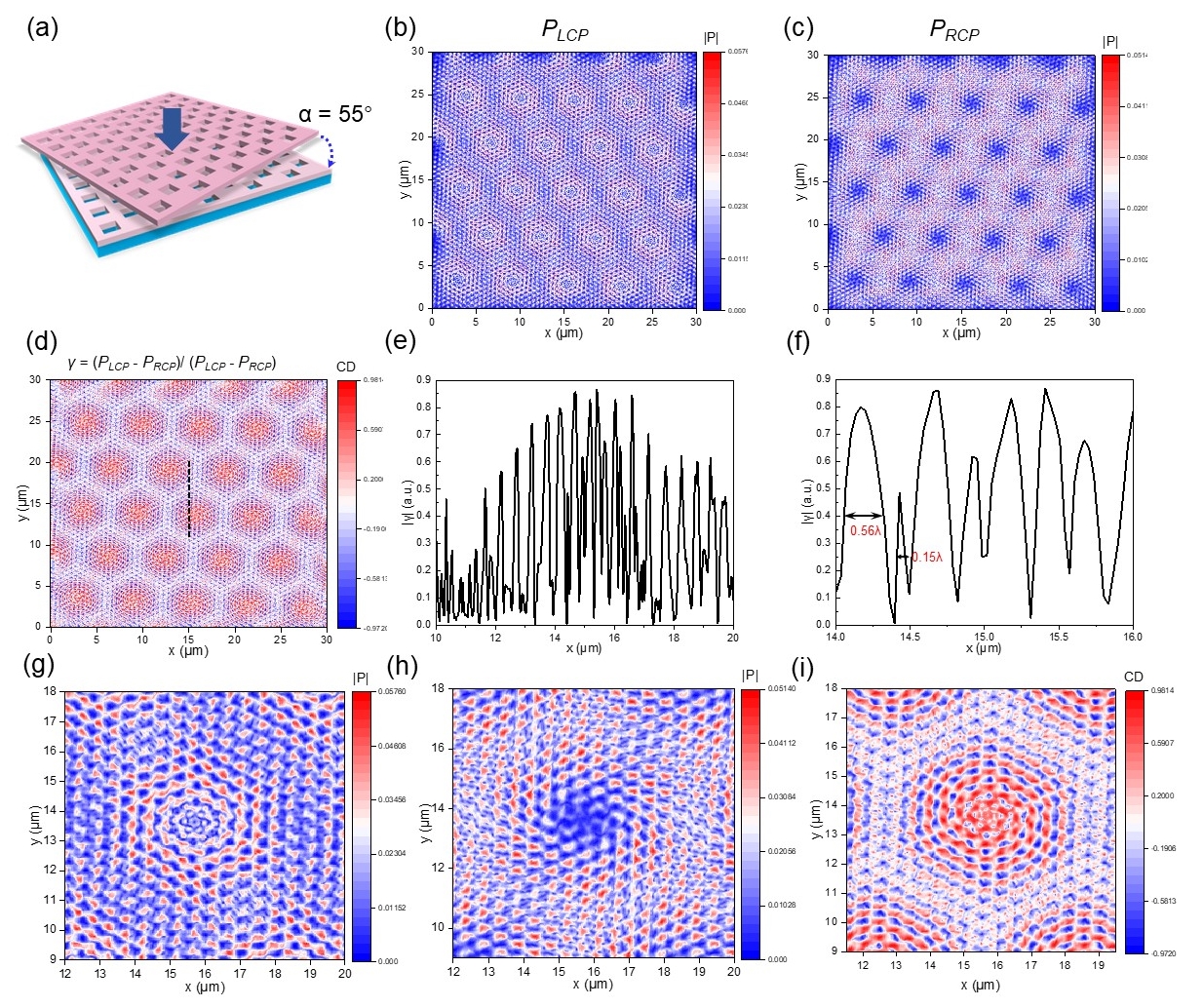


**Figure S1** Light manipulation and subwavelength features of the twisted bilayer meta-devices. (a) Schematic of the twisted bilayer meta-device. The twist angle is 55°. The wavelength of the normal incident light is 535 nm. (b, c) The magnitude of the Poynting vector under the incidence of LCP beam (b) and RCP beam (c). (d) The spatial variations of the magnitude of the Poynting vector versus the spin states are calculated as *γ* = *(P_LCP_*-*P_RCP_*)/(*P_LCP_*+*P_RCP_*). (e) A detailed view of the spatial variations of the part indicated by a black dashed line shown in (d). The absolute value of the spatial variations |γ| is shown to facilitate the quantitative characterization of the feature size. (f) A zoomed-in view of (e). (g-i) The zoomed-in view of (b), (c), and (d), respectively.

**
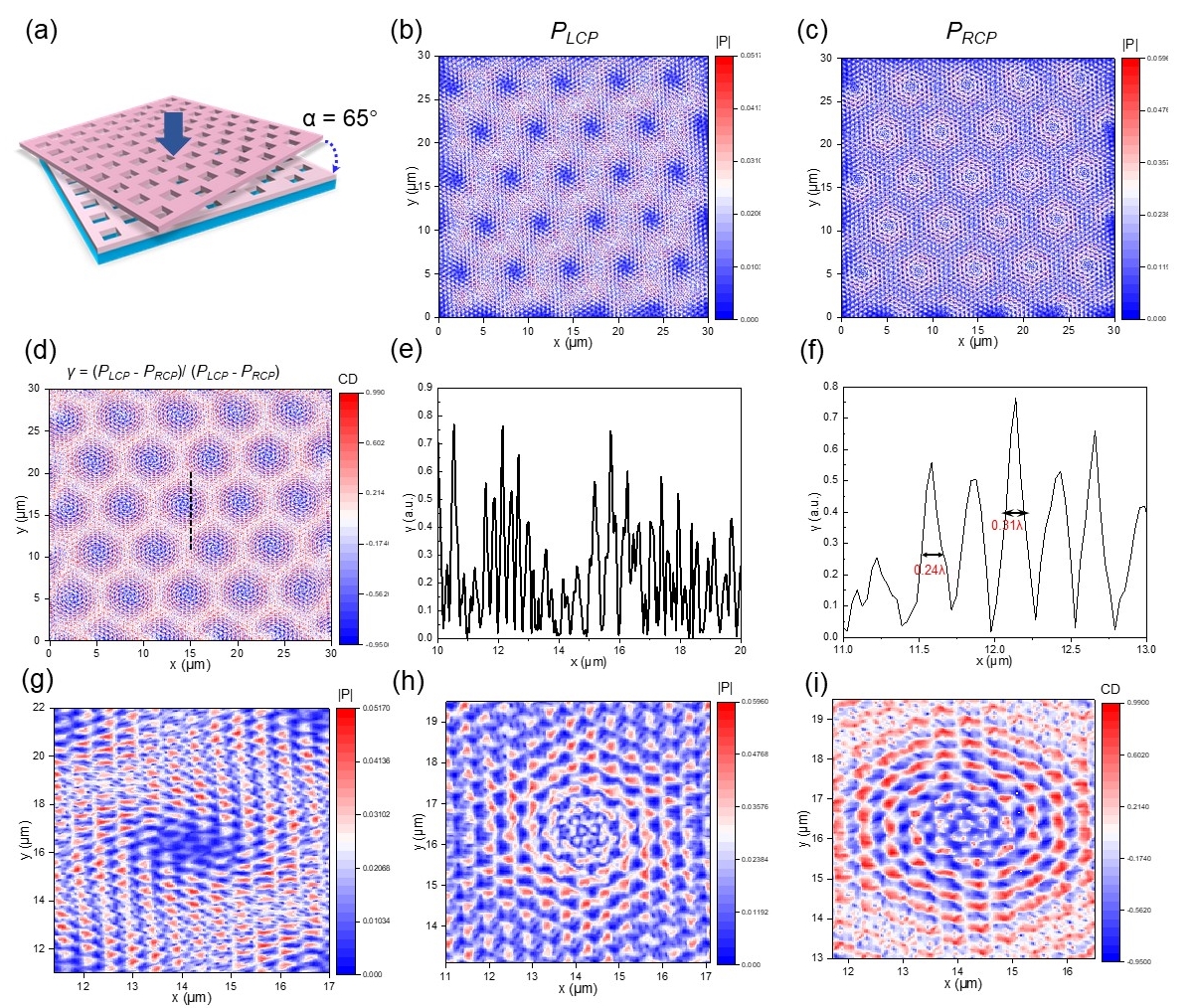
**

**Figure S2** Light manipulation and subwavelength features of the twisted bilayer meta-devices. (a) Schematic of the twisted bilayer meta-device. The twist angle is 65°. The wavelength of the normal incident light is 535 nm. (b, c) The magnitude of the Poynting vector under the incidence of LCP beam (b) and RCP beam (c). (d) The spatial variations of the magnitude of the Poynting vector versus the spin states are calculated as γ = (*P_LCP_*-*P_RCP_*)/(*P_LCP_*+*P_RCP_*). (e) A detailed view of the spatial variations of the part indicated by a black dashed line shown in (d). The absolute value of the spatial variations |γ| is shown to facilitate the quantitative characterization of the feature size. (f) A zoomed-in view of (e). (g-i) The zoomed-in view of (b), (c), and (d), respectively.

**Section 2: Period of moire pattern**

Table S1 shows the calculated and measured period of the moire pattern with various twist angles of 20.3°, 56.8°, 60.9°, and 64.4°, respectively. We calculated the period of the moire pattern by Equation (S1).

$L=\frac{A}{2\sin\frac{\Delta\theta}{2}}$ (S1)

where L is the period of the moiré pattern, A is the period of the nano-holes, and $\Delta\theta$ is the absolute degree difference between the twist angle and the closest axis of symmetry, namely, 0°, 60°, 120°, and so on. The calculated period is compared to the measurements, and they show a good agreement with the experimental results. The measured moiré pattern period was found to be 1.4, 7.3, 35.1, and 8.0 µm for twist angles of 20.3°, 56.8°, 60.9°, and 64.4°, respectively.

**Table S1** **Periods of the moiré patterns of the twisted bilayer meta-devices with different twist angles.**

| twist angles | 20.3° | 56.8° | 60.9° | 64.4° |
| --- | --- | --- | --- | --- |
| Calculated Periods | 3.2 µm | 9.7 µm | 34.4 µm | 7.0 µm |
| Measured Periods | 1.4 µm | 7.3 µm | 35.1 µm | 8.0 µm |

**Section 3: Simulation mode verification and experimental setup**

**
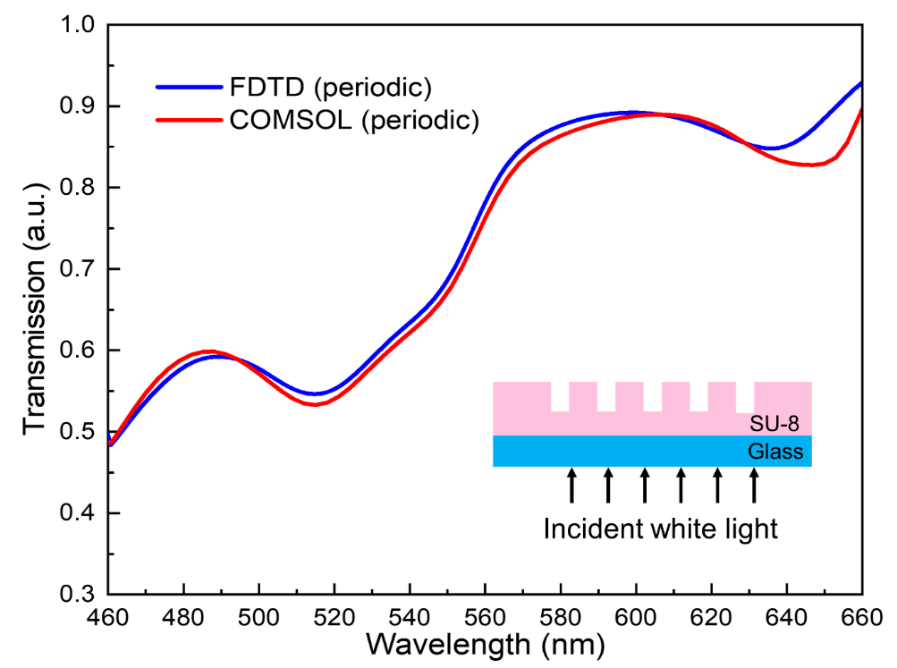
**

**Figure S3** Simulation of single-layer sample. The red and blue lines are the simulated transmission of the single-layer sample using Ansys Lumerical FDTD^®^ and COMSOL Multiphysics^®^, respectively. The PML condition is employed in the light direction, and periodic conditions are employed for the other two orthogonal directions in these two simulations. Inset shows the schematic of the model. The model is obtained by removing the top and middle layers of the twisted bilayer meta-devices. The blue part represents the glass substrate, and the pink part represents the SU-8. The thickness of the glass substrate is 500 µm, the SU-8 film is 415 nm, and the hole on the SU-8 film is 280 nm. The length of the square hole is 300 nm, and the period of the nano-hole is 535 nm. The black arrows indicate the direction of the incident light. The wavelength of the left circular polarization beam is from 460 nm to 660 nm.

**
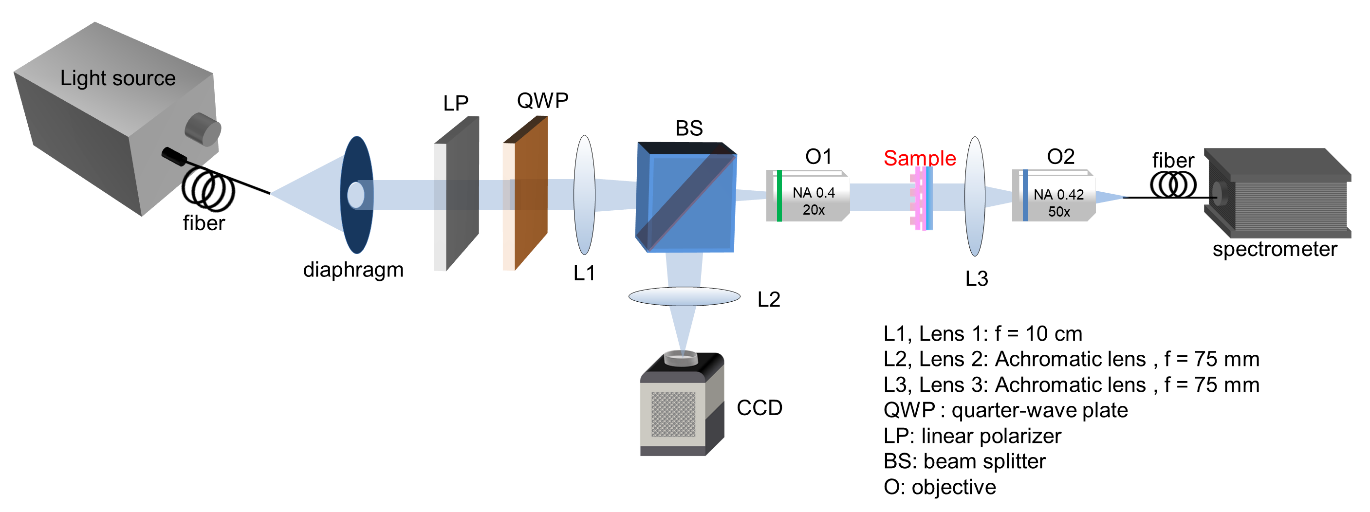
**

**Figure S4** Experimental setup of the spectrum measurement. The incident beam is generated by the light source (adjustable tungsten light source H03, Seeman Technology Co., Ltd. ), and a diaphragm is used to control the spot size of the beam incident onto the sample. The linear polarizer and the quarter-wave plate are used to generate the left/right circular polarization states (LCP/RCP). An objective (20× magnification, NA = 0.4) is used to focus the incident circular polarization beam onto the sample, and another objective (50× magnification, NA = 0.42) is used to collect the beam from the sample in transmission into the spectrometer (Maya2000 Pro, Ocean Insight). The CCD is used to locate different sample areas for multiple measurements.

**
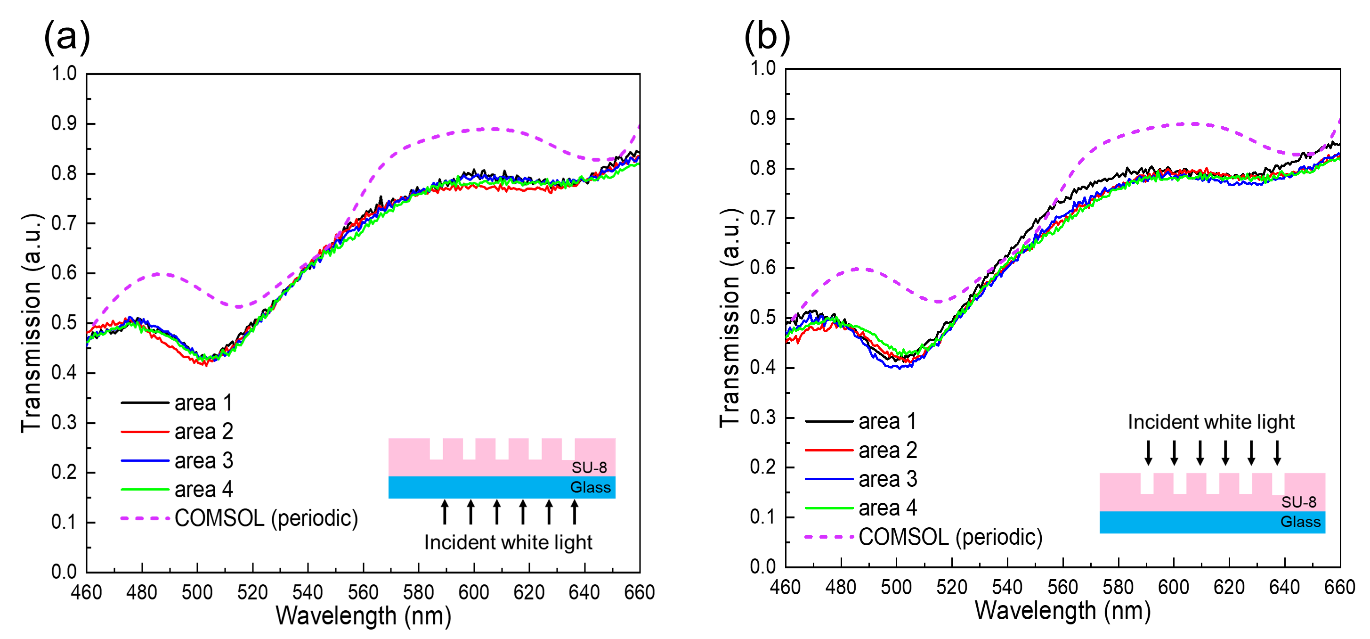
**

**Figure S5** Experimental results of single-layer sample. (a) The simulated and experimental transmission when the left circular beam is incident from the glass substrate side. (b) The simulated and experimental transmission when the LCP light incidents from the top side. In both figures, the solid lines of different colors represent the measured results of different sample areas. The dashed lines show the simulated transmission of the meta-atom using COMSOL Multiphysics^®^. The simulation setup is the same as that in Figure S2. These two inserts show the schematics of the model. The model is the same as that in Figure S2. The black arrows indicate the direction of the incident light.

**
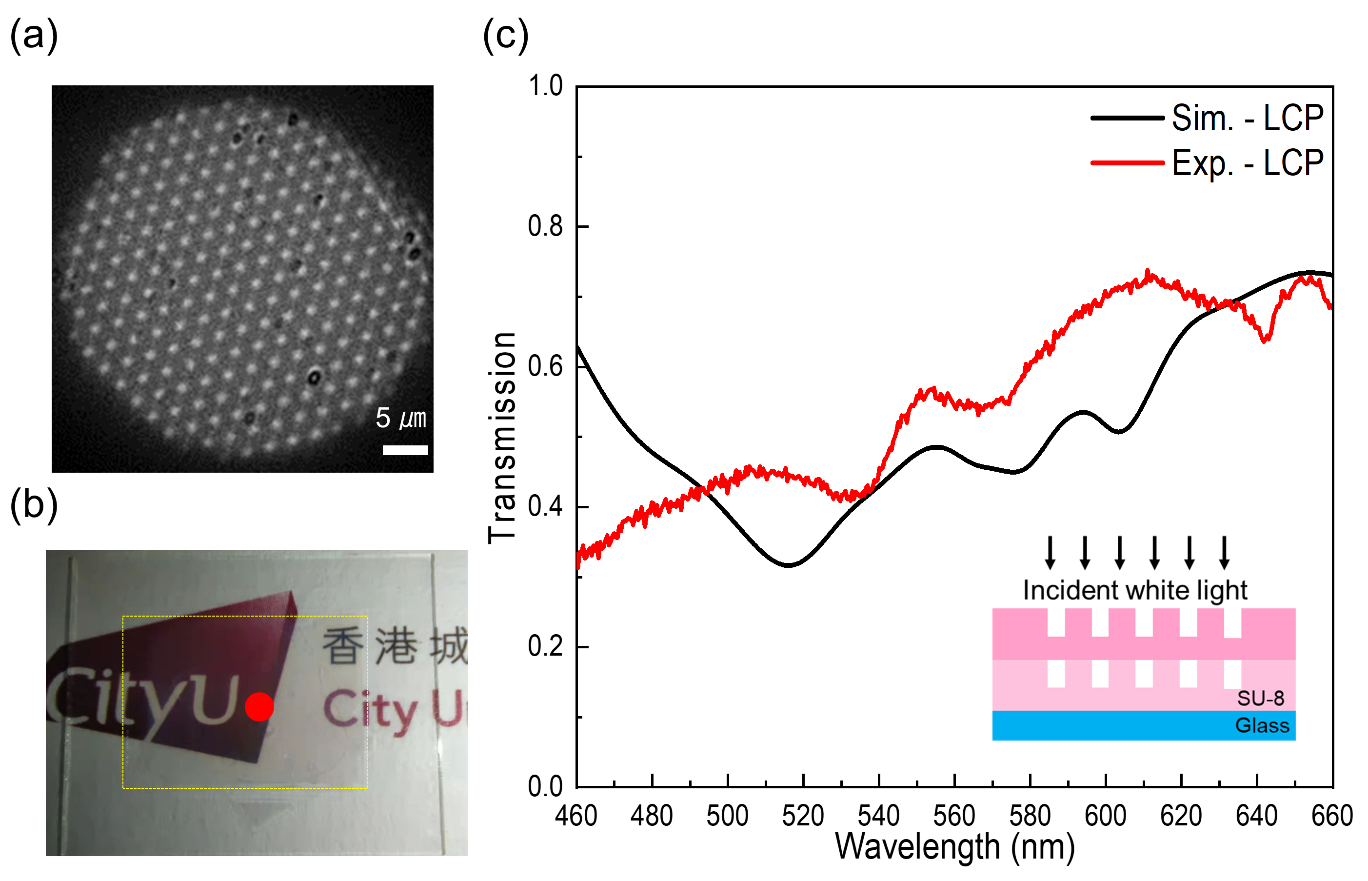
**

**Figure S6** Experimental results of twisted bilayer meta-device. (a) Optical image of the sample with a 56.8° twist angle. The scalar bar is 5 µm. (b) Optical image of the sample with a 56.8° twist angle at lower magnification. The sample with the twisted bilayer meta-devices is transparent with high transmission characteristics. The yellow dashed box is the sample area. The red circle is the area of the transmission measurement under LCP light. (c) The black and red lines show the simulated and the experimental transmission of the sample with a 56.8° twist angle. The insert shows the schematic of the twisted bilayer meta-device. The blue part represents the glass substrate, the pink part represents the SU-8 part, and dark pink and light pink represent the top layer and the bottom layer shown in Figure 1, respectively. The thickness of the two layers of the air hole is 280 nm, and the SU-8 film between them is 280 nm. Below this bottom air hole is another SU-8 film of 415 nm, and the thickness of the glass substrate is 500 µm.

**
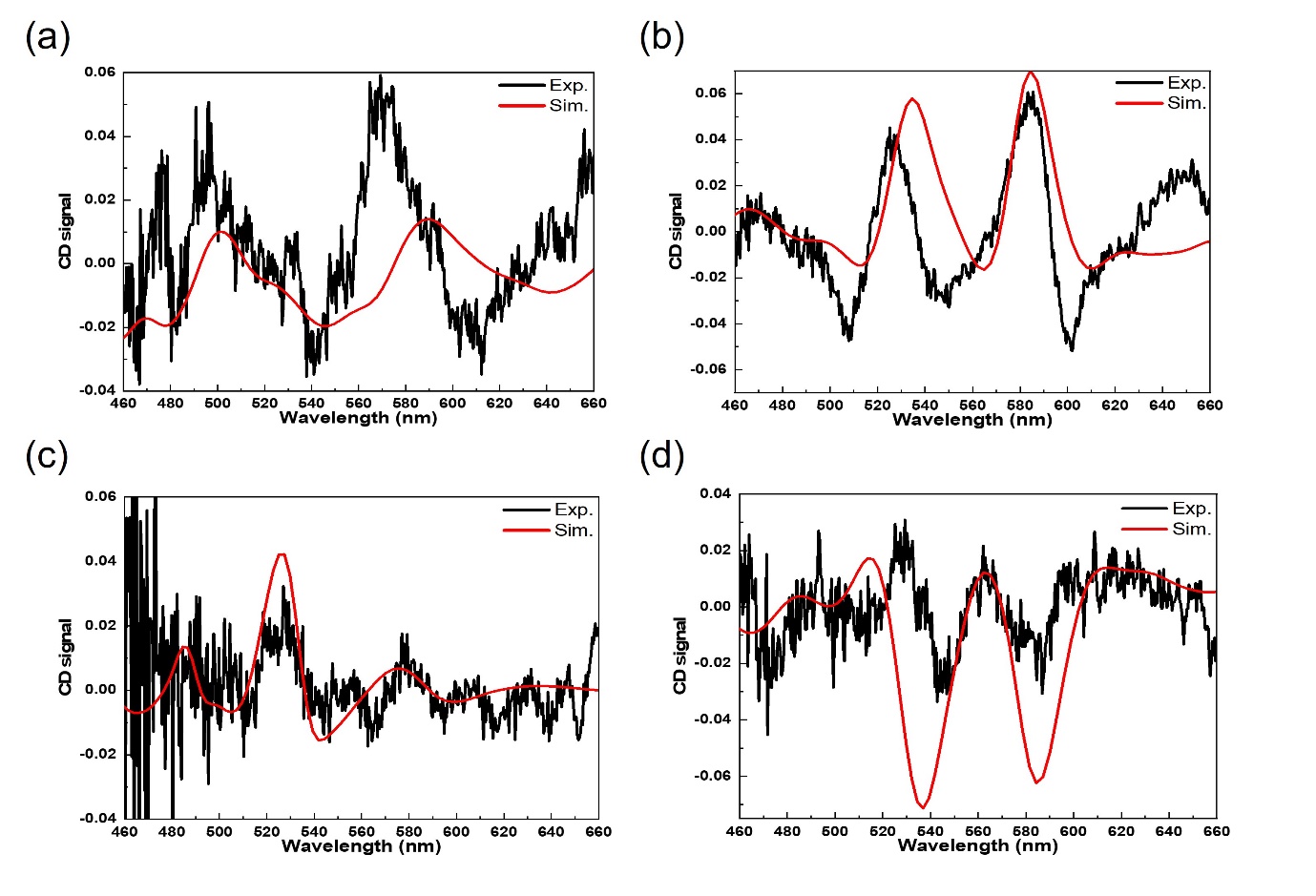
**

**Figure S7** Simulation and experimental results of CD signals. (a) Calculated (red line) and experimental (black line) CD signal of the sample with 20.3° twist angle. (b) Calculated (red line) and experimental (black line) CD signal of the sample with 56.8° twist angle. (c) Calculated (red line) and experimental (black line) CD signal of the sample with 60.9° twist angle. (d) Calculated (red line) and experimental (black line) CD signal of the sample with 64.4° twist angle. For all the calculated CD signals, we first aquired the simulated transmission of the LCP/RCP incident beam (*T_LCP_* and *T_RCP_*) using Ansys Lumerical FDTD^®^, and then we calculated the transmission contrast (*T_LCP_* -*T_RCP_*)/( *T_LCP_* + *T_RCP_*) to get the CD signal results. For all the experimental CD signals, we first measured the transmission of the LCP/RCP incident beam (*T_LCP_* and *T_RCP_*), and then we calculate (*T_LCP_* -*T_RCP_*)/( *T_LCP_* + *T_RCP_*) to get the CD signal results.

Figure S8 shows the experimental and simulated CD signals of the meta-device with the twist angle of 60.9°. The measured moire pattern period of the meta-device with a 60.9° of twist angle is about 35.1 µm (please see Table S1), so we used this twist angle as the sample. We did simulations for two simulation areas of the meta-device, 40 µm by 40 µm and 50 µm by 50 µm, respectively. The simulation results show similar spectra, which means the simulation results have become convergence. The results are still consistent with the experimental results and support our conclusion. The largest simulation area is 50 µm by 50 µm due to our computer's computing power and its calculation ability. For the other twist angle meta-devices (20.3°, 56.8°, and 64.4°), the measured moire pattern period of the meta-device is 3.2, 9.7, and 7.0 µm, respectively. The moire pattern period is much smaller than the current simulation area (20 µm by 20 µm), which is big enough for good approximations.


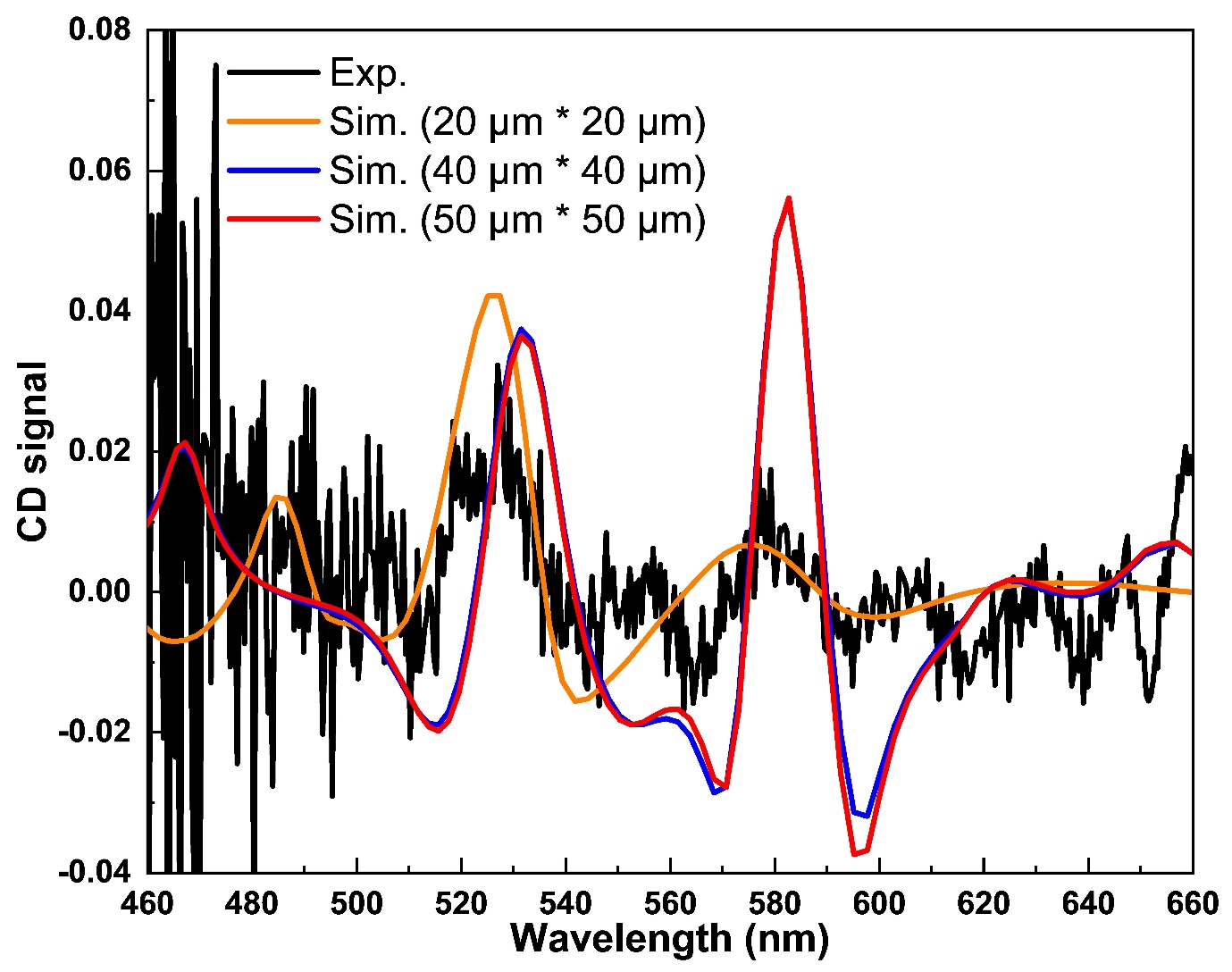


**Figure S8** The experimental and simulated CD signals of the meta-device with the twist angle of 60.9°. The black line is the experimental CD signal. The orange, red and blue lines are the simulated results. The simulation area of the orange line is 20 µm by 20 µm; the red line’s simulation area is 40 µm by 40 µm, and the red line’s simulation area is 50 µm by 50 µm.

In addition, we show simulation results for closer gap distances between the two slabs, which are 30 µm and 50 µm. The dielectric meta-device could confine light well, but the strong coupling between the two slabs needs a close gap. The thinner gap could induce extra strong coupling, leading to a stronger CD signal, as shown in Figure S9. The CD signal becomes much stronger when the gap distances are 30 or 50 µm.


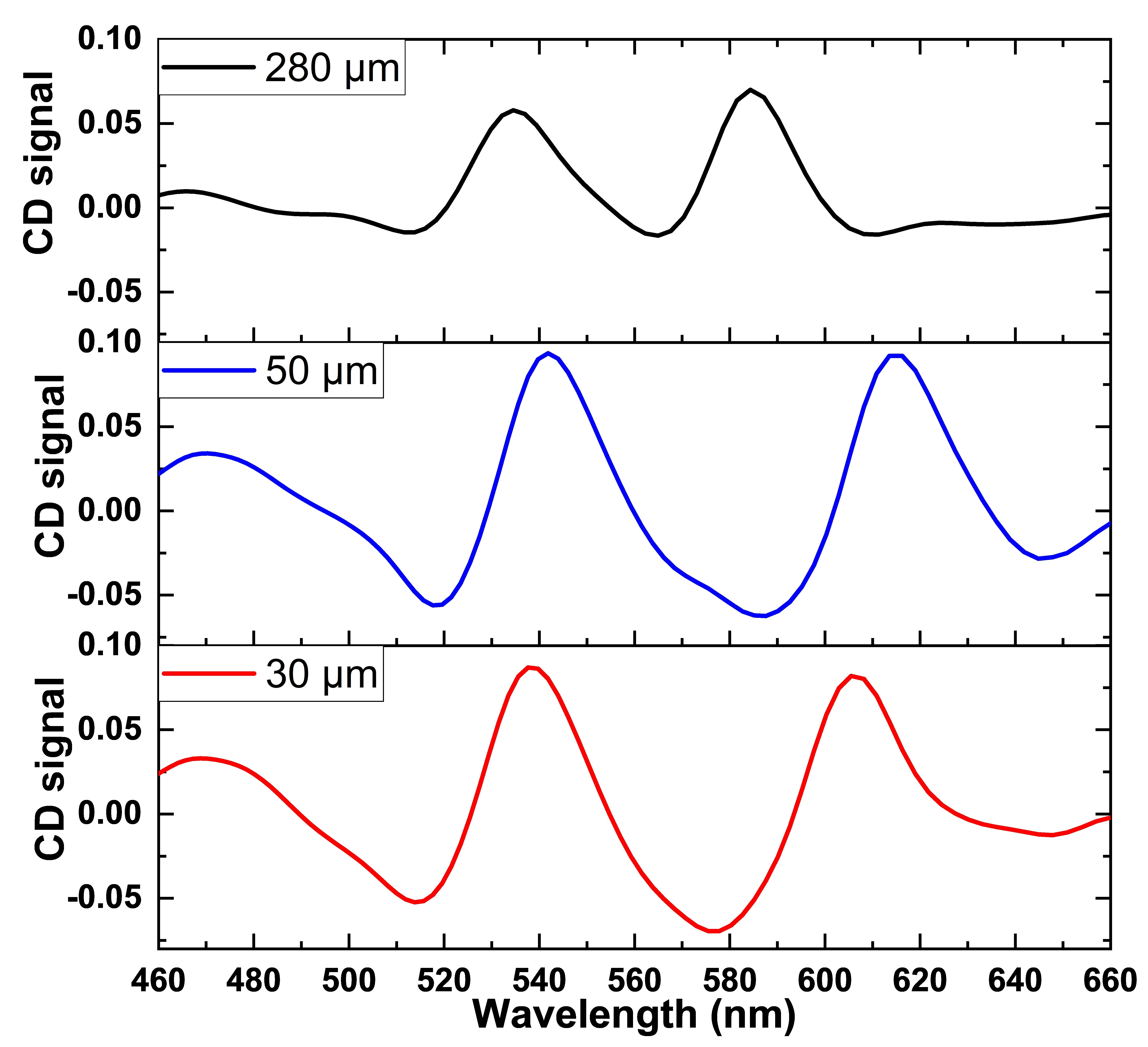


**Figure S9** Simulated CD signals versus different distances between two slabs when the twist angle is 56.8°. Here are the simulated CD signals when the gap between the two layers is 30, 50, and 280 µm, respectively.

Figure S10 shows simulation results using the rounded square hole shape of the fabricated nanoholes. The twist angle of the simulations is 20.3°. Since the simulation for a structure with rounded squares consumes much more computer resources, our computer can only support the simulation area of up to 6 µm by 6 µm. The simulation results show that the CD signals of the square and rounded square are similar and match the experimental result.


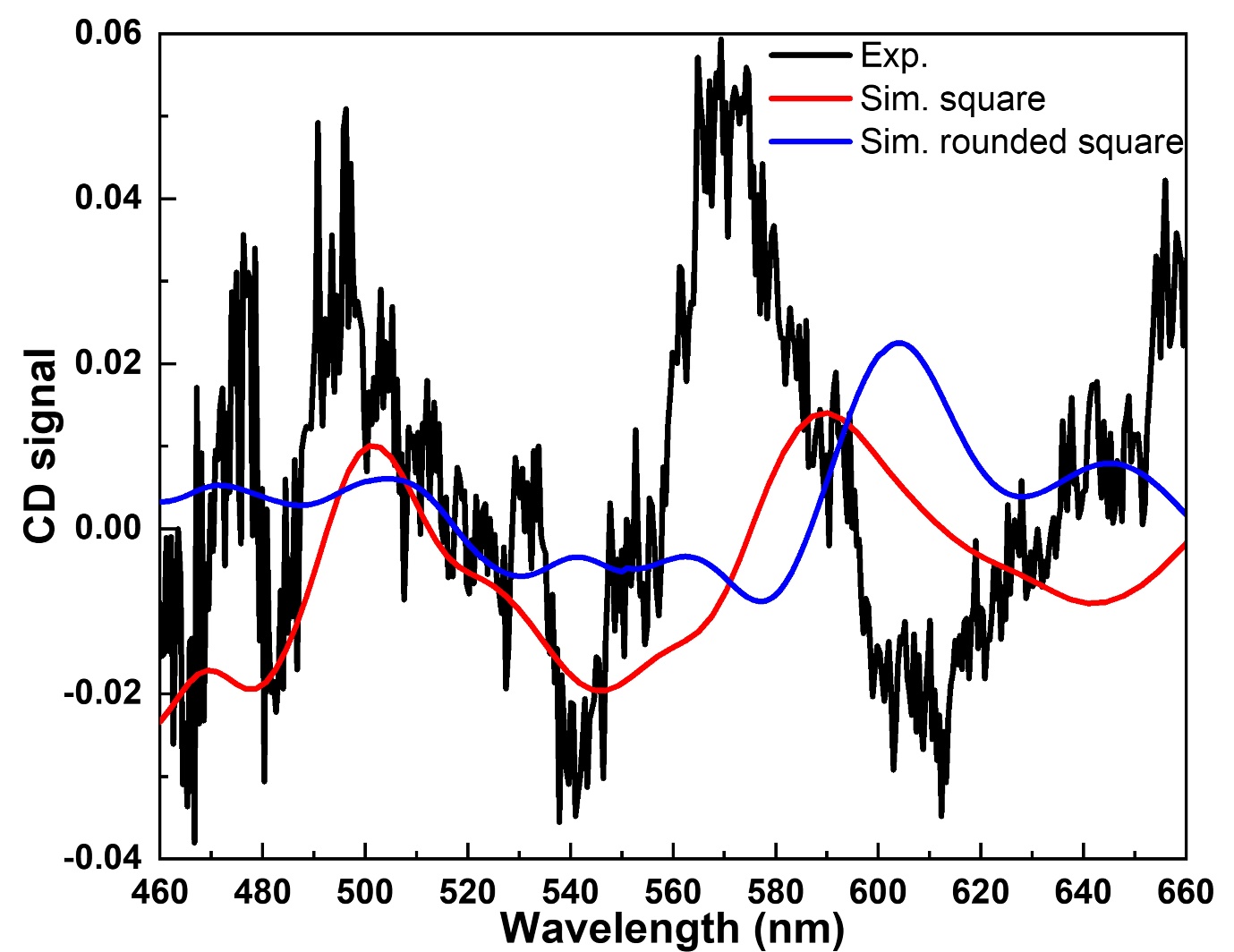


**Figure S10** CD signals when the twist angle of the meta-device is 20.3°.The black line is the experimental result. The red line is the simulated result. The nanohole in the model is square, the same as our design. The blue line is also the simulated result, but the nanohole in the model is built as the fabricated sample, which is a rounded square.

**Section 4: Chiral magic angle**

**
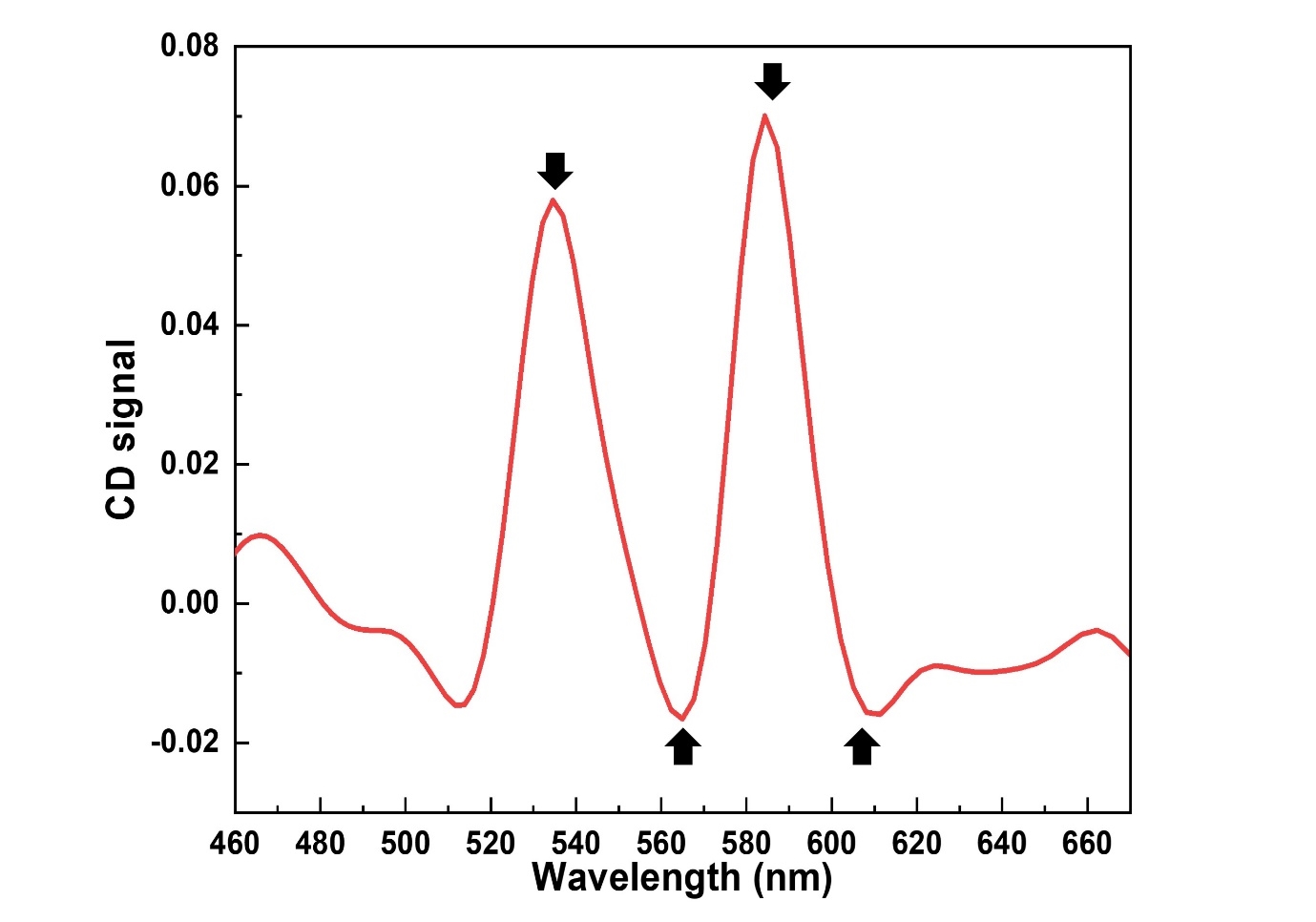
**

**Figure S11** Selected wavelength for the figure plotting of the chiral magic angle. The simulated CD signal of the twist bilayer meta-device with the twist angle of 56.8°. The four arrows indicate the four peak/dip values of the CD signal. We select these four wavelengths (534.5 nm, 564.9 nm, 587.2 nm, 608.2 nm) to provide proof for the chiral magic angle in Figure 6.

**
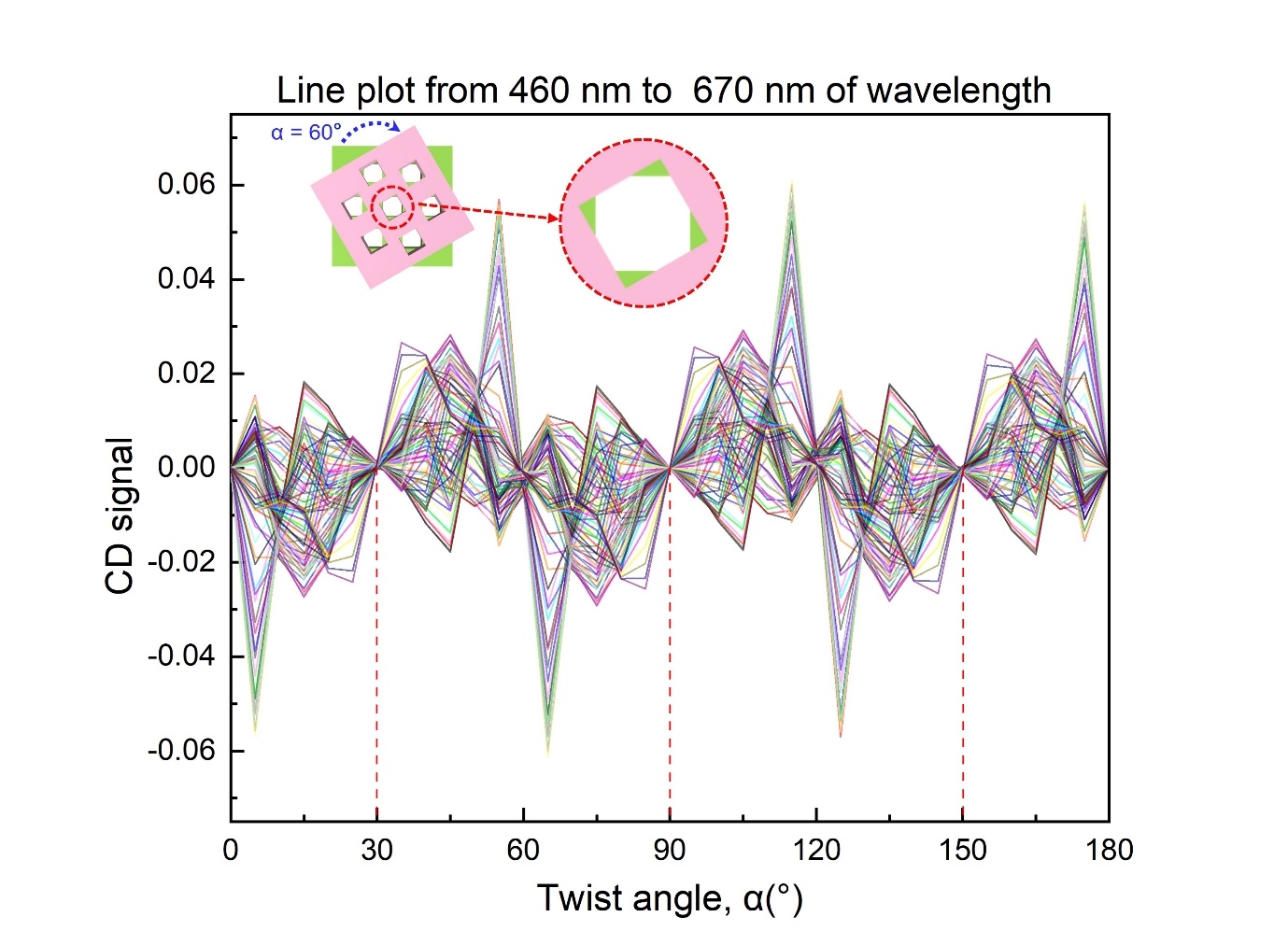
**

**Figure S12** Chiral magic angle of the square-like nano-hole of the twisted bilayer meta-device. The colored lines are the CD signals, and they cover the spectrum ranging from 460 nm to 670 nm. The chiral magic angles occur at 30°, 90°, and 150° twist angles. The inset shows a schematic of the twisted bilayer meta-device with a twist angle of 60°. The zoomed-in image shows the existence of an imperfectly symmetrical structure. The imperfect symmetry introduces different optical responses to LCP and RCP light. Therefore, the CD signals are slightly changed.


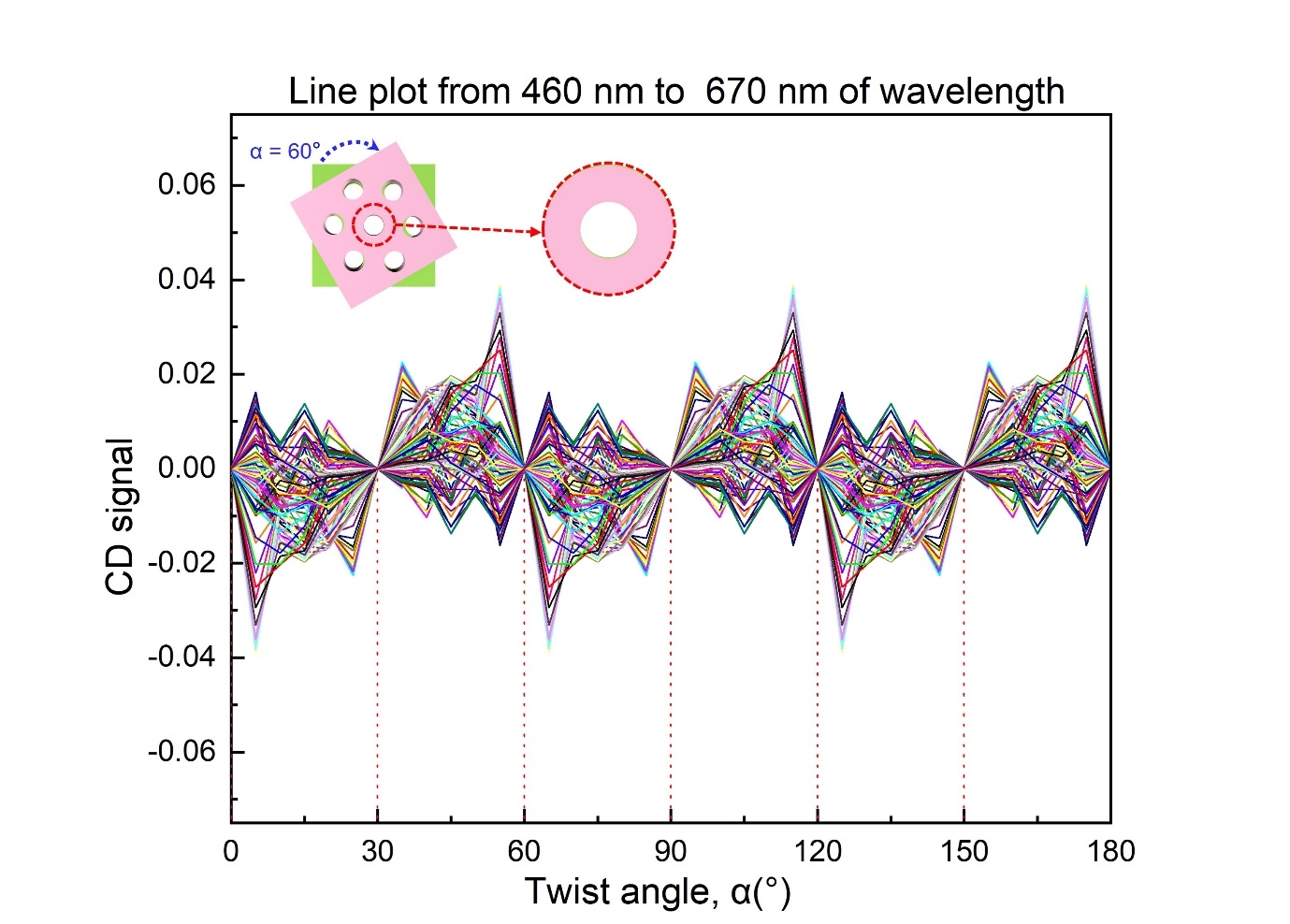


**Figure S13** Chiral magic angle of the circular-like nano-hole of the twisted bilayer meta-device. The colored lines are the CD signals, and they cover the spectrum ranging from 460 nm to 670 nm. The chiral magic angles appear at every 30° of twist angle. The insert shows the schematic of the twisted bilayer meta-device with a twist angle of 60°. The zoomed-in image shows the perfect symmetry structure, which is polarization-insensitive. Compared with Figure S8, the twist angles of 60° and 120° become the chiral magic angles.

Figures S14-1 and S14-2 show different shapes of meta-atoms of the twisted bilayer meta-devices. The circular hole of a 300 nm diameter (a), a square with a side length of 300 nm (b), and rectangular holes with a side length ranging from 310-450 nm (c - j) are shown, respectively. The width of these rectangles is fixed at 300 nm. The first column is a schematic of the structure. The second column is the corresponding CD signal. The third, fourth, and fifth columns are calculated to describe the symmetry axis of the 30°, 60°, and 90° axis. We use Figure S14-1(a) as an example to illustrate our calculation method. For the symmetry axis of 30°, in the CD signal in Figure S14-1 (a), the two parts, 0-30°, and 30-60° are anti-symmetric. If we add the CD signals of the twisted bilayer meta-devices with the twist angles of 0° and 60°, these two CD signals will cancel each other. The same applies to the CD signals of 5° and 55°, 10° and 50°, 15° and 45°, and so on, for they are the anti-symmetric groups. Finally, we obtain a zero matrix. 0-30° and 60-90° are translational symmetric, so if these two parts are subtracted, a zero matrix will also be obtained. For the symmetry axis of 60°, the two parts, 0-60° and 60-120°, are translational symmetric, so subtracting these two parts will also result in a zero matrix. It similarly applies to 0-90° and 90-180° parts. If this symmetry or anti-symmetry is broken, then the calculated matrix will not be zero. This value can be used to describe and compare the degree of symmetry breaking. In a similar way, the above calculation is performed for the other shapes of meta-atoms, and the corresponding 30°, 60° and 90° symmetry matrices are obtained. It shows that the values in the 30° and 60° symmetry matrix get larger and larger while the 90° symmetry matrix stays at zero when the shape of the nanoholes is changed from circular, square, to rectangular. This indicates that the symmetry and anti-symmetry at 30° and 60° are weakened, while the symmetry at 90° remains the same when the shape of the nanoholes is changed. The calculated value of the standard deviation for the 30°, 60°, and 90° symmetry axis are shown in Figure 7(e), respectively.

**
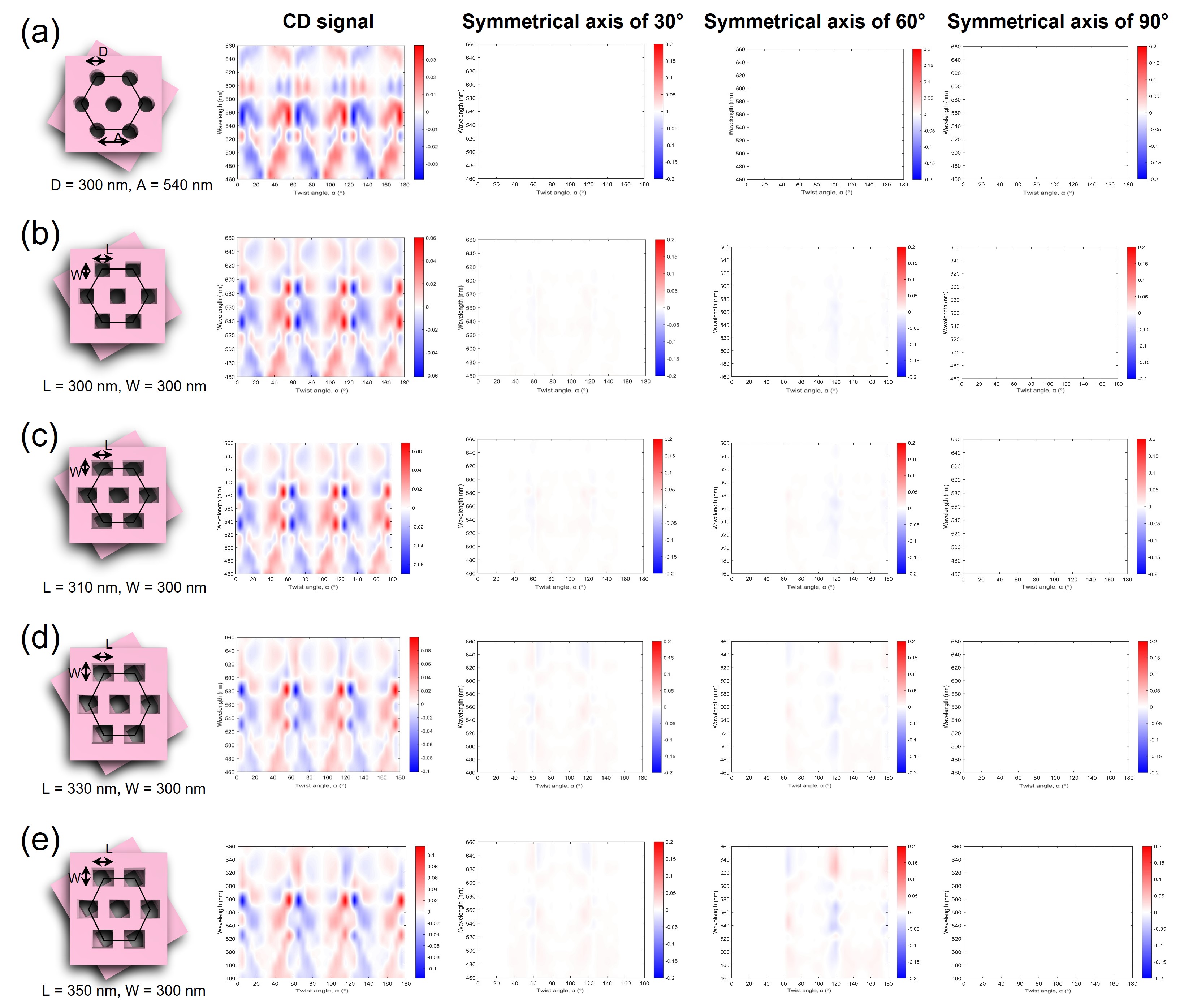
**

**Figure S14-1** Chiral magic angle of the twisted bilayer meta-device with various shapes of meta-atoms. The meta-atoms are (a) circular holes with 300 nm of diameter, (b) square holes with 300 nm of side length, and (c-e) rectangular holes with a fixed width of 300 nm and various lengths of 310 to 350 nm.

**
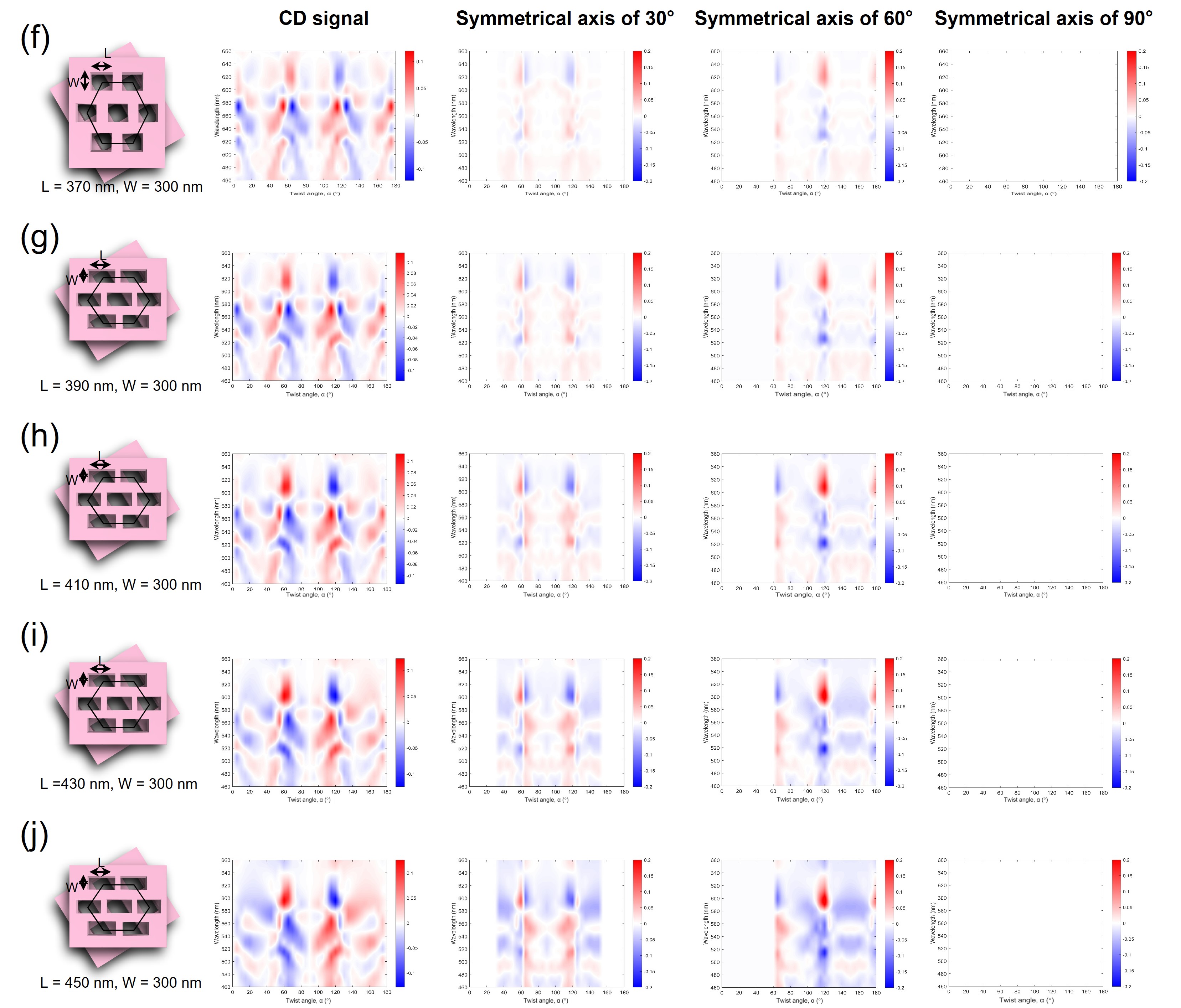
**

**Figure S14-2** Chiral magic angle of the twisted bilayer meta-device with various shapes of meta-atoms. The meta-atoms are (f-j) rectangular holes with a fixed width of 300 nm and various lengths of 370 to 450 nm.
